# Electrochemistry Facilitates the Chemoselectivity of Benzylic Alcohol Oxidations Mediated by Flavin-Based Photoredox Catalysis

**DOI:** 10.1021/acs.orglett.5c04603

**Published:** 2026-01-27

**Authors:** Rostislav Sponar, Alan Liška, Radek Cibulka

**Affiliations:** † Department of Organic Chemistry, University of Chemistry and Technology, Prague, Technická 5, 166 28 Prague 6, Czech Republic; ‡ Department of Molecular Electrochemistry and Catalysis, J. Heyrovský Institute of Physical Chemistry ASCR, Dolejškova 3, 18223 Prague 8, Czech Republic

## Abstract

The use of oxygen for catalyst regeneration in photoredox catalysis causes selectivity problems, because the generation of reactive oxygen species initiates overoxidations and side oxidations. Herein, we present a system using the electrochemical regeneration of a flavin catalyst under inert conditions. Our process allows the chemoselective oxidation of primary and secondary benzylic alcohols to carbonyl compounds without unwanted overoxidation to carboxylic acids. The method tolerates other oxidation-sensitive groups such as methyl, methylsulfanyl, or pinacolboryl groups.

## Graphical abstract

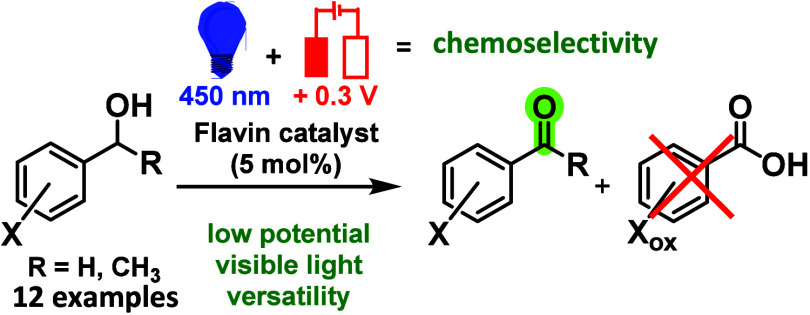

The selective oxidation of benzylic alcohols to carbonyl compounds is an important chemical process because of the extensive use of the target compounds in pharmaceutical and chemical industries.^1^ The methods that provide this transformation require selectivity, but their potential for adverse environmental impacts also needs serious consideration.^2^ Therefore, the original procedures, which used toxic transition metal-containing stoichiometric reagents,^3^ are now being replaced by metal-free reagents,^4^ electrochemistry,^5^ or catalytic processes using oxygen as the oxidizing agent.^6^ Another recent alternative is photoredox catalysis, which uses the oxidizing power of a catalyst excited by visible light, thereby providing environmentally friendly benzyl oxidations.^7^ However, these photoredox methods have limited chemoselectivity, as oxidation to carbonyl compounds is often accompanied by undesired overoxidation to carboxylic acids or side oxidation of an easily oxidizable group. Consequently, few highly selective photocatalytic systems are available for benzyl oxidations.^7b,8^

In recent decades, flavins have emerged as outstanding photocatalysts for visible light-mediated reactions.^9^ One of the most significant flavin representatives is the naturally occurring vitamin B_2_ (riboflavin), whose acetylated derivative—riboflavin tetraacetate (**RFTA** (Figure 1A))—is widely used in photooxidations.^10^ Despite its many advantages, such as maximum absorption in the blue region and easy access, the use of **RFTA** is still limited due to its excited state oxidation potential of 1.67 V,^11^ which is a relatively low value, especially for the oxidation of demanding substrates. This obstacle might be overcome by the coordination of **RFTA** to metal ions^11,12^ or by using flavinium salts **1**.^10b,13^ However, the selectivity and effectiveness of photocatalytic oxidation reactions with flavin derivatives suffer from the need to use oxygen as a regenerating agent (Figure 1B). Oxygen in photooxidations often causes the formation of reactive oxygen species (ROS), which are then responsible for undesired oxidations and catalyst deactivation.^14^ This issue could possibly be resolved by applying regenerating agents under an inert atmosphere. The regenerating role of acetonitrile has recently been demonstrated for benzyl alcohol photooxidations with deazaflavinium salts (Figure 1C).^8a,15^

**Figure 1:**
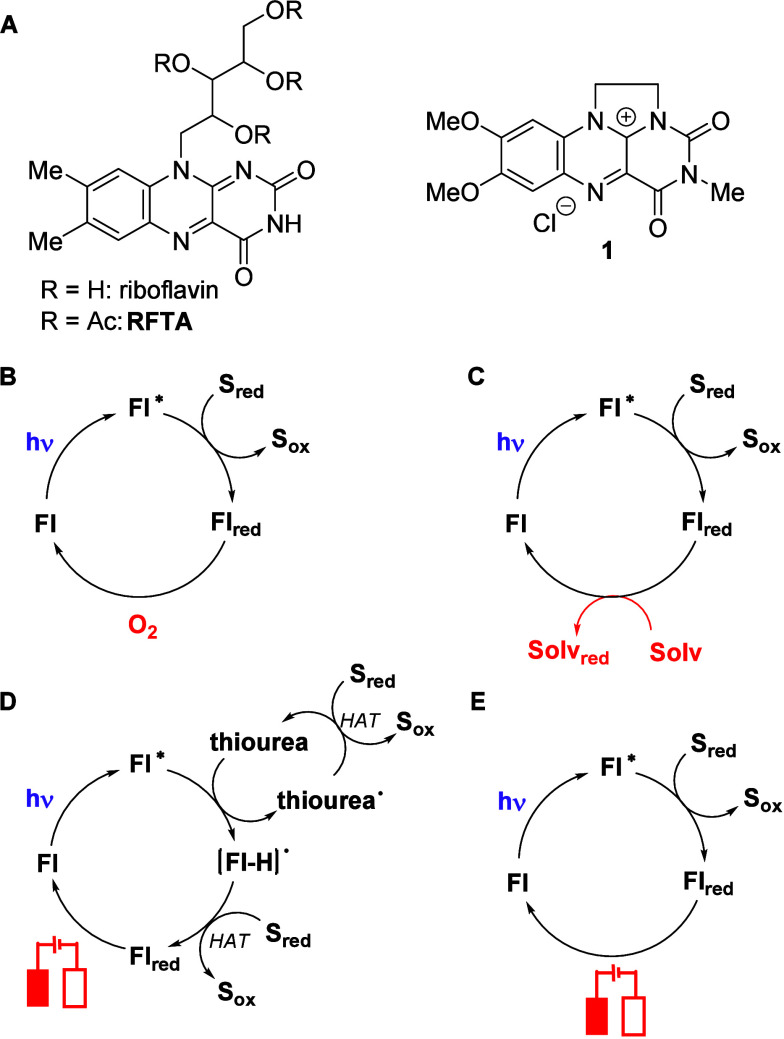
Structures of the selected flavin catalysts (A) and the simplified catalytic cycles representing photooxidations of a substrate (**S**) with flavin derivatives (**Fl**) under aerobic conditions (B) and using solvent (Solv) as the sacrificial oxidant^8a,15^ (C). Oxidation by using electrochemical flavin (**Fl**) regeneration with thiourea as a cocatalyst^17b^ (D) and under additive-free conditions (E; this work).

A combination of electrochemistry and photochemistry (electrophotocatalysis)^7c,16^ offers a solution to the oxygen problem in oxidation reactions by providing regeneration of the catalyst via electrochemical oxidation at the anode. Nevertheless, this approach is not yet widely used.^16,17^ In the field of flavin photooxidations, Lin et al. recently published a photoelectrochemical oxidative system using a combination of an **RFTA** catalyst with thiourea.^17b^ This system oxidizes less reactive alcohols via hydrogen atom transfer to thiyl radicals generated by the action of RFTA* (Figure 1D). In our paper, we present a simple electrophotochemical method using flavin derivative **1** as a single catalyst, without additives (Figure 1E). Because of the electrochemical regeneration of the catalyst and the oxygen-free conditions, the method allows the chemoselective transformation of benzyl alcohols to carbonyl derivatives without overoxidation or side oxidation.

For the initial development of our method, we used **RFTA** as the catalyst and acetonitrile as a solvent, analogously to the previously reported photooxidations with flavin catalysts,^13b^ keeping in mind that acetonitrile is a suitable solvent for electrochemistry. However, we also tested an acetonitrile/water (9:1, v/v) mixture, since adding water is known to suppress the susceptibility of flavins to aggregation.^18^ Based on preliminary experiments (Table S1), we chose the following electrochemical setup: platinum mesh as the working electrode, platinum wire as the counter electrode, silver wire as the reference electrode, and tetrabutylammonium hexafluorophosphate (TBAPF_6_) as the electrolyte. Oxidations were carried out in an electrochemical cell irradiated with a 450 nm LED in an argon atmosphere for 8 h (see the section S3 of the Supporting Information for details). The potential was set to 0.3 V with regard to the ground state redox potentials of the flavin catalysts.^19^ 4-Methoxybenzyl alcohol (**2a**) (*E*_ox_ = 1.60 V)^20^ was used as a model substrate. In addition, 4-chlorobenzyl alcohol (**2b**) (*E*_ox_ = 2.16 V)^13a^ was selected as a representative of “more difficult” substrates with higher oxidation potentials. We note that the analytical scale experiments were performed in deuterated solvents for easier monitoring by ^1^H NMR.

Experiments with **RFTA** showed that the acetonitrile/water mixture was a more suitable solvent (cf. entry 1 vs entry 2 and entry 3 vs entry 4, Table 1). In contrast to the oxidation of **2a** (entries 1 and 2), **RFTA** was ineffective in the oxidation of **2b** (entries 3 and 4). Therefore, we turned our attention to the more powerful ethylene-bridged flavinium salts. Specifically, we selected dimethoxy derivative **1**, which exhibits high photostability while maintaining a relatively high oxidation power (*E*_red_* = 2.4 V).^13b^ Our experiments showed that **1**, especially in the acetonitrile/water mixture, exhibits high efficiency in the oxidation of both **2a** and **2b** (entries 5–8). Moreover, in all oxidations under electrophotochemical conditions, aldehyde **3** was formed as a single product, and we did not observe overoxidation to carboxylic acid **4**. In contrast, aerobic photooxidation using oxygen as a sacrificial oxidant generated a significant amount of acid **4** (entries 9–12).

**Table 1:**
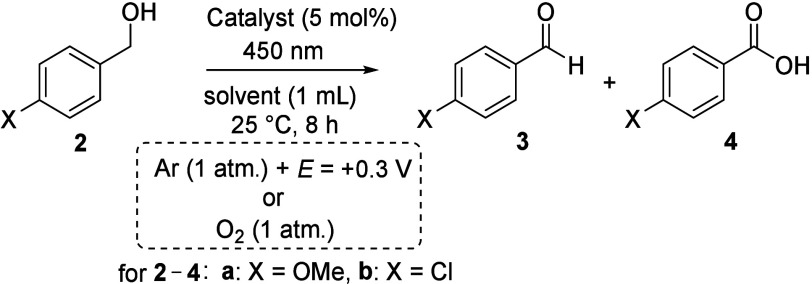
First Proof of Chemoselective Electrophotochemical Oxidation with Flavins^a^,^b^

					yield (%)^c^	
entry	catalyst	atmosphere	X	solvent	**3**	**4**
1	**RFTA**	Ar	OMe	CD_3_CN	37	0
2	**RFTA**	Ar	OMe	CD_3_CN/D_2_O	49	0
3	**RFTA**	Ar	Cl	CD_3_CN	11	0
4	**RFTA**	Ar	Cl	CD_3_CN/D_2_O	20	0
5	**1**	Ar	OMe	CD_3_CN	22	0
6	**1**	Ar	OMe	CD_3_CN/D_2_O	82	0
7	**1**	Ar	Cl	CD_3_CN	25	0
8	**1**	Ar	Cl	CD_3_CN/D_2_O	77	0
9^e^	**1**	O_2_	OMe	CD_3_CN	72	37
10^e^	**1**	O_2_	OMe	CD_3_CN/D_2_O	79	19
11^e^	**1**	O_2_	Cl	CD_3_CN	36	11
12	**1**	O_2_	Cl	CD_3_CN/D_2_O	38	6

a Selected data; for further experiments, see Table S2.

b Conditions: catalyst (5 μmol), **2** (0.1 mmol), solvent (1 mL), 450 nm, 25 °C, argon, 8 h, TBAPF_6_ (0.1 M), *E* = 0.3 V, Pt mesh as the working electrode, Pt wire as the counter electrode, Ag wire as the reference electrode.

c Determined by ^1^H NMR.

d At a 9:1 (v/v) ratio.

e Oxygen (balloon) instead of argon, without an applied potential.

We further optimized the developed system using a mixture of substrates: 4-chlorobenzyl alcohol (**2b**) and thioanisole (**5**). The latter is known to be easily oxidized by various flavins to the corresponding sulfoxides under aerobic conditions.^21^ This mixture was intended to mimic a substrate with different oxidizable groups. Moreover, we wanted to exclude possible photosulfoxidation with water (component of the solvent system) as a source of the oxygen molecule.^22^ When tested with a mixture of substrates **2b** and **5** (Table 2), the electrophotocatalytic system proved to be effective and provided aldehyde **3b** exclusively; thioanisole (**5**) remained unreacted in the reaction mixture (entry 1), which is in contrast to “classical” aerobic photooxidation giving a mixture of products such as **4b**, **6**, and **7** (entry 2; see section S3.3 for details). Further attempts to change the electrophotochemical system did not lead to any improvements. The reaction proceeded less efficiently with other electrolytes, such as tetramethylammonium hexafluorophosphate (TMAPF_6_) and tetrabutylammonium tetrafluoroborate (TBABF_4_) (entries 3 and 4, respectively), in other solvents, such as DMF-*d*_6_ and CDCl_3_ (entries 5 and 6, respectively), or with higher and lower applied potentials (entries 7 and 8, respectively). In accordance with previous experiments, the system with **RFTA** was less efficient (entry 9), like that with flavin **8** possessing a trifluoromethyl group (entry 10). The activity of flavinium salt **9** was similar to that of **1a** (entry 11); we chose salt **1** because of its higher photostability. Control experiments confirmed that the developed system needs a catalyst (entry 12), an applied potential (entry 13), and light (entry 14).

**Table 2:**
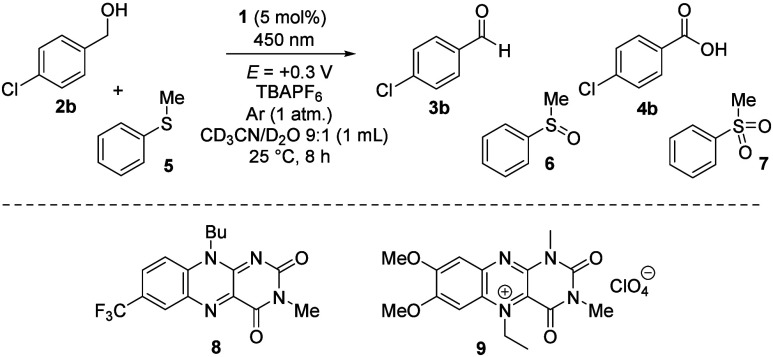
Competitive Experiments of Electrophotochemical Oxidation of **2b** in the Presence of **5** ^a^

		yield (%)^b^			
entry	variation from the standard conditions	**3b**	**4b**	**6**	**7**
1	no	64	0	0	0
2	photoredox system^c^	58	5	97	trace
3	TMAPF_6_ as the electrolyte	50	0	0	0
4	TBABF_4_ as the electrolyte	35	0	0	0
5	DMF-*d*_6_ as the solvent	5	0	0	0
6	CDCl_3_ as the solvent	8	0	0	0
7	*E* = 0.15 V	63	0	0	0
8	*E* = 0.45 V	65	0	0	0
9	RFTA as the catalyst	14	0	0	0
10	**8** as the catalyst	16	0	0	0
11	**9** as the catalyst	64	0	0	0
12	no catalyst	0	0	0	0
13	no potential	trace	0	0	0
14	no light	0	0	0	0

a Conditions: **1** (5 μmol), **2b** (0.05 mmol), **5** (0.05 mmol). For other details, see Table 1.

b Determined by ^1^H NMR.

c Oxygen (balloon) instead of argon, without an applied potential.

Using the optimized procedure, we performed a series of preparative experiments on a 1 mmol scale (Figure 2). In the case of primary benzyl alcohol **2**, oxidations provided the corresponding aldehyde as the only product. In the cases with less than quantitative conversions, the unreacted material remained in the reaction mixture. The oxidation of benzyl alcohols substituted at the *para* position with electron-donating (OMe and Me) or weakly electron-withdrawing substituents (Cl) occurred with quantitative or high conversions, and the corresponding aldehydes **3a**–**3c** were isolated in good yields.

**Figure 2:**
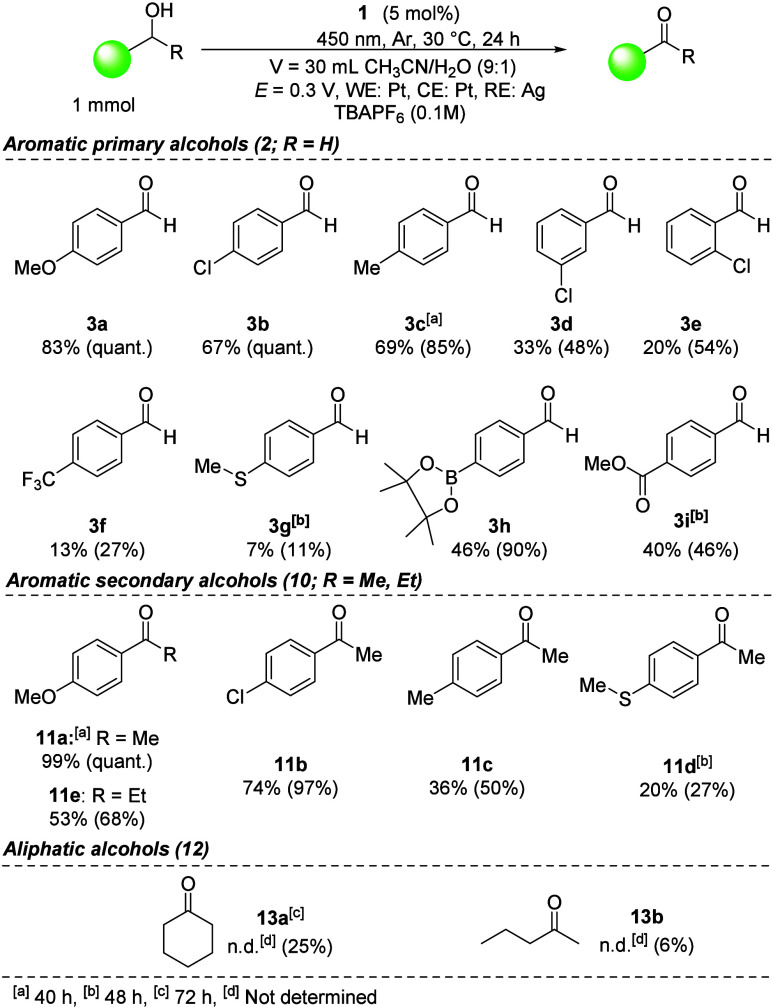
Conversions (^1^H NMR yields in parentheses) and preparative yields of electrophotooxidations on a 1 mmol scale (see the Supporting Information for details).

The applicability of the method for both *meta*- and *ortho*-substituted derivatives is demonstrated in chloro derivatives **3d** and **3e**. A lower yield was obtained in the case of strongly electron-deficient trifluoromethyl derivative **3f** that forms the limit of applicability of our method due to the very high oxidation potential of the corresponding alcohol **2f** (*E*_ox_ = 2.70 V).^13a^ A less difficult substrate with a methoxycarbonyl group gave aldehyde **3i** in good yield.

Interestingly, the yield of hydroxy group oxidation in the case of the methylsulfanyl derivative **2g** was relatively low. This may be because intermediary radical cation **2g** ^•+^ (Figure 3) has a relatively high electron density on the sulfur atom. This radical is expected to readily convert into the corresponding sulfoxide in the presence of oxygen, whereas the reaction toward the aldehyde is less productive. Also, the elimination of a hydrogen atom from radical **2** ^•+^ is more difficult for **2g** than for the methoxy (**2a**), chloro (**2b**), or methyl (**2c**) derivatives because of the higher bond dissociation energy (section S6). Contrary to **3g**, a good yield and high conversion were achieved in the case of pinacol phenylboronate **3h**, where benzyl alcohol **2h** was efficiently oxidized to the aldehyde without affecting the pinacolboryl group. This group is easily oxidized by conventional oxidation methods to form phenol.^23^ In fact, this transformation occurs even with UVA light irradiation in the presence of an amine^24^ or during photooxidation of **2h** with **1** under aerobic conditions (Table S6).

**Figure 3:**
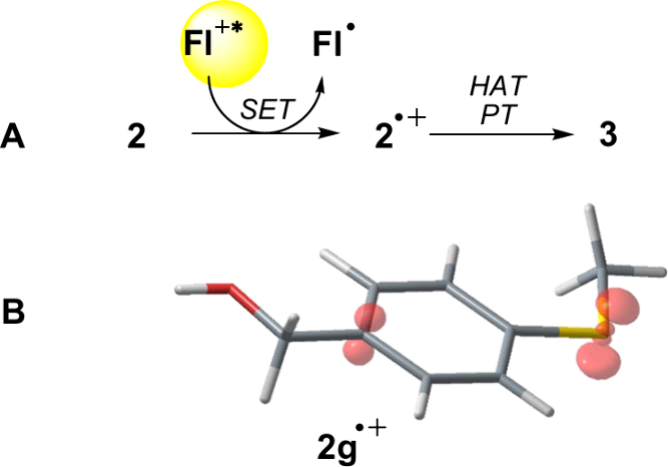
(A) Proposed transformation of alcohol **2** to aldehyde **3**. (B) Visualized spin electron density for intermediate **2g** ^•+^ calculated by DFT. For details on calculations, see sections S6 and S7.

The oxidation of secondary benzyl alcohols **10** produced exclusively ketones **11**, with no undesirable oxidation of oxidizable groups, such as methyl in **11c** or sulfur in **11d**. The reactions gave ketones in excellent to good yields, except for methylsulfanyl derivative **11d**. Oxidation also proceeded with aliphatic substrates **12**, but with low conversions to ketones **13**, reflecting the high oxidation potential.

Based on the study of aerobic photooxidations with flavin catalysts,^1a,13b^ we propose a mechanism for the new electrophotochemical oxidation. Upon photoexcitation of flavinium catalyst **1**, an electron is transferred from the substrate to the catalyst in a singlet excited state^13b^ to form **Fl** ^**•**^ and a radical cation of an alcohol (Figure 3A). The thermodynamic realness of SET is confirmed by the appropriate potentials (see above) and by a fluorescence quenching experiment (section S5). Subsequently, a hydrogen atom and proton are transferred to form a carbonyl compound. The flavin is then regenerated by electrochemical oxidation at the anode. The potential of 0.3 V is high enough to oxidize both fully reduced flavin and flavin radical to **Fl** ^**+**^ (*E*_1_ = −0.44 V; *E*_2_ = −0.97 V vs SCE; see ref 19 and section S4).

In summary, we have found a suitable method for the chemoselective oxidation of primary and secondary benzyl alcohols to carbonyl compounds. This method allows us to exploit the strong oxidation ability of flavinium salt **1** excited by visible light, but because of the electrochemical regeneration of the catalyst, it does not cause the overoxidation of carboxylic acids or the unwanted oxidation of other groups, such as methylsulfanyl, methyl, or pinacolboryl groups. Our approach also demonstrates that a combination of photoredox catalysis and electrochemistry is an effective strategy for increasing the chemoselectivity of reactions, especially dehydrogenations, which do not require an oxygen source.

## Supplementary Material



## Data Availability

The data underlying this study are available in the published article, in its Supporting Information, and openly available in Zenodo at DOI 10.5281/zenodo.17523965.

## Notes

Experimental details about the synthesis and characterization of substrates, electrophotooxidation methods, extended data on method optimization and mechanistic studies, details on theoretical calculations, characterization of oxidation products, and copies of NMR spectra (PDF)
