# Supplementary material for: Electrochemistry Facilitates the Chemoselectivity of Benzylic Alcohol Oxidations Mediated by Flavin-Based Photoredox Catalysis
Source: Org Lett. 2026 Jan 27;28(5):1540–4. doi: 10.1021/acs.orglett.5c04603 (PMC12888019; doi:10.1021/acs.orglett.5c04603)
Supplement: Supplementary file 1 [file ol5c04603_si_001.pdf]

Supporting Information

Electrochemistry facilitates the chemoselectivity of benzylic alcohol oxidations mediated by flavin-based photoredox catalysis

Rostislav Sponar, Alan Liška, Radek Cibulka*

Content

S1. Materials and instrumentation 2

S2. Preparation and characterisation of starting alcohols 3

S3. Electrophotocatalytic dehydrogenations of alcohols 4

S3.1 General procedure A – analytical scale (0.1 mmol) 4

S3.2 General procedure B – semipreparative scale (1 mmol) 4

S3.3 Optimization of conditions 5

S3.4 Preparation and characterization of isolated products 9

S3.5 NMR spectra of isolated products 12

S3.6 Comparison of NMR spectra of experiments conducted under O_2 and Ar atmosphere 28

S4. Spectroelectrochemical data 29

S4.1 Electrochemical redox potential of catalysts 29

S4.2 UV stability of photocatalyst in the mixture with substrates 30

S4.3 Electrochemical spectra 32

S5. Fluorescence quenching study 33

S6. Theoretical calculations 34

S7. Cartesian coordinates of the optimized structures 37

S8. References 49

S1. Materials and instrumentation

Chemicals were purchased at Sigma-Aldrich and Fluorochem. Flavin derivatives were prepared according to a procedure described in literature.[1-6] The solvents were purified and dried using standard procedures. Commercially obtained reagents were used as received without further purification unless otherwise stated. Thin layer chromatography (TLC) analyses were carried out on DC Alufolien Kieselgel 60 F254 (Merck). The compounds were visualised with UV light (254 and 366 nm). Flash chromatography was carried out using Büchi Pure C-810 at Silica 40 µm irregular column at increased pressure. Compound structures were drawn and named using ChemDraw.

Nuclear magnetic resonance (NMR) spectra were recorded in CD_3CN on an Agilent 400-MR DDR2 (399.94 MHz for ^{1}H, 100.58 MHz for ^{13}C, 376.50 MHz for ^{19}F), JNM-ECZL400G spectrometer JEOL Ltd., (399.94 MHz for ^{1}H, 100.58 MHz for ^{13}C, 376.50 MHz for ^{19}F) or JNM-ECZL400G spectrometer JEOL Ltd., (399.94 MHz for ^{1}H, 100.58 MHz for ^{13}C, 376.50 MHz for ^{19}F) at 298 K unless otherwise indicated. Data for ^{1}H NMR are reported as follows: chemical shift (δ ppm), multiplicity (s = singlet, d = doublet, t = triplet, q = quartet, m = multiplet, dd = doublet of doublets, dt = doublet of triplets, br = broad etc.), coupling constant (Hz), and integration. All NMR spectra were processed and assigned using MestreNova. **High-resolution mass spectra** were obtained on Q-Tof Micro (Waters), equipped with a quadrupole and time-of-flight (TOF) analyser and a multichannel plate (MCP) detector. The melting points were measured on a Boetilus melting point apparatus and are uncorrected.

Absorption spectra were recorded on a UV-VIS HP8452 (Hewlet Packard) spectrophotometer at 25 °C in analytical-grade solvents. **Emission spectra** were recorded on a Varian CARY ECLIPSE fluorescence spectrophotometer at 25 °C in analytical-grade solvents.

Spectro-electrochemistry measurements were performed in an OTTLE Cell (Optically transparent thin-layer electro-chemical cell)[7], pathlength = 0.02 cm, working electrode: Pt minigrid, counter electrode: Pt: minigrid, pseudo reference electrode: Ag wire. Samples were prepared by degassing a solution via argon bubbling for several minutes. UV-Vis spectra were recorded on Agilent Cary 8454 spectrometer. **Cyclic voltammetry** (CV) experiments were carried out with an Autolab PGSTAT302N Metrohm, working electrode: glassy Carbon (Metrohm, 3 mm GC disk RDE electrode); counter electrode: Platinum sheet (ca. 1 cm^2); pseudo reference electrode: Silver wire (Metrohm, anhydrous); supporting electrolyte: tetrabutylammonium tetrafluoroborate (Fluka), 0.1 M in CH_3CN solution was degassed with argon in a titration vessel (10 – 90 mL, Metrohm). Ferrocene was used as an internal reference. Diamond paste was used as polishing material. Absorption, emission spectra and cyclic voltammograms were processed by using Microsoft Excel and Origin 2018 (OriginLab).

Photochemical Setup: The reaction setups for the photocatalysis were the same as described in our previous work.[1] Reactions were performed in vessels from borosilicate glass using commercial LED(s) as a light source: Luxeon LED Engin, 1.35 W@700 mA, 450 nm (dominant wavelengths 435-460 nm).

Electrophotochemical Setup: The analytical setups for the electrophotocatalysis were performed in cuvette from borosilicate glass, working electrode: Platinum mesh; counter electrode: Platinum wire; pseudo reference electrode: Silver wire (Xi'an Yima Optoelec); supporting electrolyte: tetrabutylammonium hexafluorophosphate (Fluorochem), 0.1 M in dry solvent was degassed with argon. The semipreparative setups for electrophotocatalysis were performed in double-jacketed electrochemical cell from borosilicate glass (Laborxing, V = 50 mL), working electrode: Platinum mesh 60 (Laborxing), Platinum wire spiral (l = 50 cm, ø 1.0 mm for each spiral S_{tot} = 15.7 cm^2, Merck), or glassy Carbon (IKA-Werke GmbH und Co); counter electrode: Platinum wire (ø 0.3, 1.0 mm, Merck); reference electrode: Silver wire (ø 0.3 mm, Merck); supporting electrolyte: tetrabutylammonium hexafluorophosphate (Fluorochem). For analytical and semipreparative setups were used commercial LEDs as a light source: Luxeon LED Engin, 1.35 W@700 mA, 450 nm (dominant wavelengths 435-460 nm).

S2. Preparation and characterisation of starting alcohols

Preparation of 1-(4-(methylthio)phenyl)ethan-1-ol (10d):

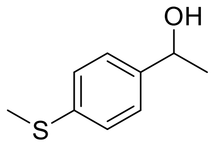

10d

Secondary benzyl alcohol **10d** was prepared according to the literature.[8] Analytical data matched that reported. Solution of $NaBH_4$ (0.55 g, 14.4 mmol, 1.2 equiv.) in 15 mL of MeOH was cooled to 0 °C under an Ar atmosphere. Keton **11d** (2.0 g, 12.0 mmol) and 15 mL of MeOH were added to $NaBH_4$ solution. After addition, the reaction mixture was stirred for 1 h at room temperature, and the course of the reaction was controlled by TLC (hexan/EtOAc – 2:1). The Reaction was quenched by the addition of saturated NH_4Cl (15 mL), and the mixture was extracted three times with EtOAc (3 × 30 mL). The organic layer was dried over Na_2SO_4 and concentrated to give a white oil, which resulted in white powder (1.57 g, 78 %) that was used without further purification, m.p.: 47-49 °C.

^{1}H NMR (400 MHz, $CDCl_3$) δ 7.30 (d, *J* = 8.6 Hz, 2H), 7.24 (d, *J* = 8.6 Hz, 2H), 4.88–4.86 (m, 1H), 2.48 (s, 3H), 1.84 (d, *J* = 3.4 Hz, 1H), 1.48 (d, *J* = 6.9 Hz, 3H).
^{13}C NMR (101 MHz, $CDCl_3$) δ 142.7, 137.4, 126.8, 126.0, 70.0, 25.1, 16.0.

The measured spectra are in agreement with literature.[9]

S3. Electrophotocatalytic dehydrogenations of alcohols

a) b) c)

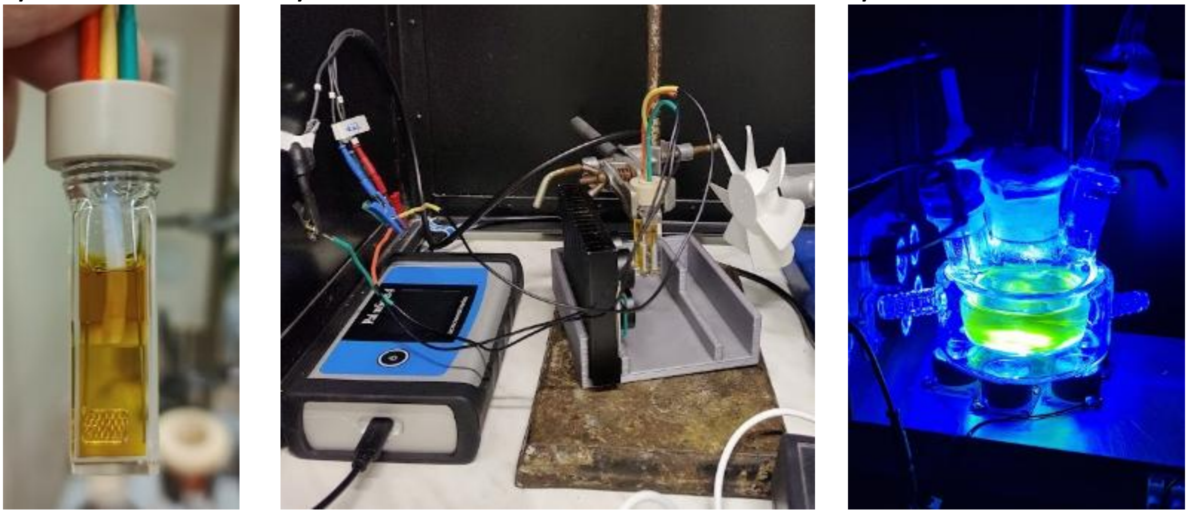

Figure S1. Experimental setup for the electrophotocatalytic dehydrogenations of alcohols under blue light irradiation (λ_{exc} = 450 nm) on analytical (a, b) and semipreparative (c) scale.

S3.1 General procedure A – analytical scale (0.1 mmol)

A benzyl alcohol/thioanisole (0.1 mmol, 1 equiv., ca. 94mM), photocatalyst (5 mol%, ca. 5mM), and supporting electrolyte $TBAPF_6$ (38.74 mg, ca. 0.1M) were dissolved in dry solvent (1 mL), and the mixture was degassed by the freeze-pump-thaw technique (3 × 5 min, p = 0.16 mbar). The reaction mixture was then transfer to electrochemical cell in glovebox. The reaction mixture was irradiated at 450 nm (Luxeon LED Engin, 1.35 W@700 mA) at a distance ca. 2 cm and was connected to potentiostat, and electrophotocatalysis was conducted at constant electric potential E = 0.3 V. The reaction time was 8 h, if not mentioned differently, using the setup shown in Figure **S1a**, **b**. The conversion of the reaction was monitored by ^{1}H NMR spectra.

S3.2 General procedure B – semipreparative scale (1 mmol)

A supporting electrolyte $TBAPF_6$ (1.16 g, ca. 0.1 M) was degassed in electrochemical cell (three-electrode system – working electrode: Platinum wire ø 1.0 mm, counter electrode: Platinum wire spiral ø 1.0 mm, pseudoreference electrode: Silver wire) under vacuum and Ar atmosphere before use. Distance between working and counter electrodes was ca. 0.5 cm. Then it was dissolved in 30 mL of mixture dry CH_3CN and H_2O in ratio 9:1. Ethylene-bridged flavinium salt **1** (0.018 g, 5 mol%, ca. 2 mM), and benzyl alcohol (1 mmol, 1 equiv., ca. 33 mM) were added. The reaction mixture was irradiated at 450 nm by three light sources (15 × 1.5 W Luxeon LED Engin 450 nm) at a distance ca. 1 cm, was connected to potentiostat, and electrophotocatalysis was conducted in potentiostatic mode at constant electric potential E = 0.3 V at 30°C. The reaction time was 24 h, if not mentioned differently, using the setup shown in Figure **S1c**. The course of the reaction was monitored by the current in time. After irradiation, the reaction mixture was evaporated and crude product was purified by flash column chromatography (hexane/EtOAc, 10 – 33% of EtOAc, v/v) to afford the corresponding aldehyde/ketone.

S3.3 Optimization of conditions

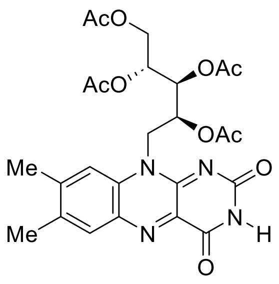

RFTA

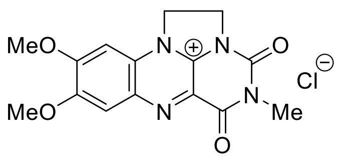

1

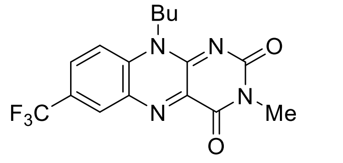

8

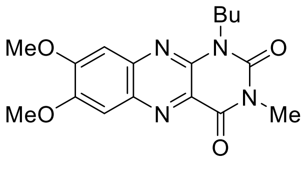

S1

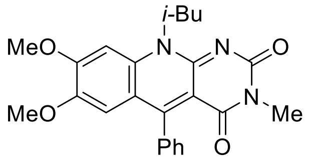

S2

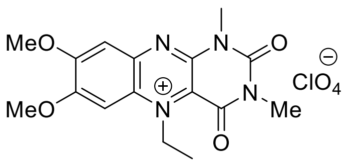

9

Figure S1. Flavin organocatalysts were prepared according to published procedures.[1-6]

Table S1. Optimization data for the semipreparative electrophotooxidation of (4-methoxyphenyl)methanol (**2a**) by photocatalysts **RFTA** and **1** according to **procedure B**

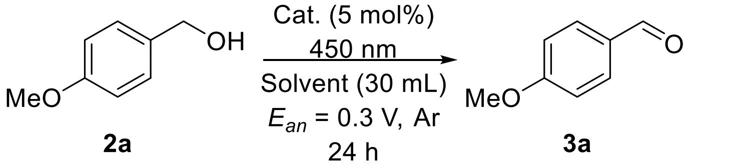

Entry	Catalyst	Substrate [mmol]	WE	CE	T [°C]	Solvent	Conversion[a]/ Yield
1	**RFTA**	2.9	Pt mesh 60	Pt (ø 0.3 mm)	25	CH_3CN	12% (11%)
2	**RFTA**	2.9	Pt mesh 60	Pt (ø 1.0 mm)	30	CH_3CN	12%
3	**1**	2.9	Pt mesh 60	Pt (ø 0.3 mm)	25	CH_3CN	18%
4	**RFTA**	1.0	Pt (ø 1.0 mm)	Pt (ø 1.0 mm)	30	CH_3CN	35%
5	**RFTA**	1.0	Pt mesh 60	Pt (ø 1.0 mm)	60	CH_3CN	46%[b]
6	**RFTA**	1.0	Pt (ø 1.0 mm)	Pt (ø 1.0 mm)	60	CH_3CN	60%[b] (37%)
7	**RFTA**	1.0	Pt (ø 1.0 mm)	Pt (ø 1.0 mm)	30	CH_3CN	30%[c]
8	**RFTA**	1.0	Pt (ø 1.0 mm)	Pt (ø 1.0 mm)	30	CH_3CN/D_2O (9:1)	63%[c] (52%)
9	**RFTA**	0.5	Pt mesh 60	Pt (ø 1.0 mm)	30	CH_3CN	64%
10	**RFTA**	1.0	Glassy carbon	Pt (ø 1.0 mm)	30	CH_3CN	35% (33%)
11	**1**	1.0	Pt (ø 1.0 mm)	Pt (ø 1.0 mm)	30	CH_3CN/D_2O (9:1)	quant.[c] (83%)

[a] Determined by ^{1}H NMR, [b] 11 × 1.5 W LED 450 nm, [c] 15 × 1.5 W LED 450 nm

Table S2. Comparison of photooxidation under oxygen atmosphere and electrophotooxidation of primary benzyl alcohols **2a** and **2b** according to **procedure A**

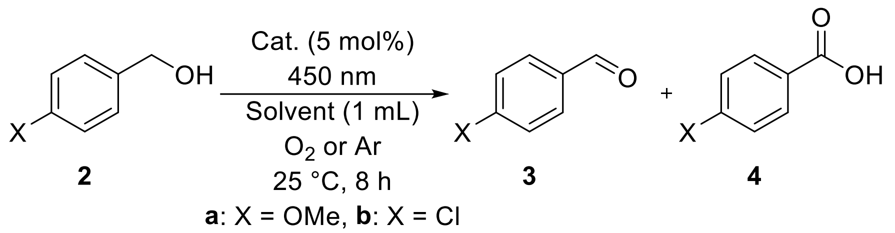

Entry	Catalyst	X	Solvent	Atm.	Conversion [%][b]	
					3	4
**1**	**RFTA**	OMe	CD_3CN	O_2	33	2
**2**	**RFTA**	OMe	CD_3CN/D_2O (9:1)	O_2	64	4
**3**	**RFTA**	Cl	CD_3CN	O_2	15	1
**4**	**RFTA**	Cl	CD_3CN/D_2O (9:1)	O_2	36	2
**5**[c]	**RFTA**	OMe	CD_3CN	Ar	37	0
**6**[c]	**RFTA**	OMe	CD_3CN/D_2O (9:1)	Ar	49	0
**7**[c]	**RFTA**	Cl	CD_3CN	Ar	11	0
**8**[c]	**RFTA**	Cl	CD_3CN/D_2O (9:1)	Ar	20	0
**9**	**1**	OMe	CD_3CN	O_2	72	37
**10**	**1**	OMe	CD_3CN/D_2O (9:1)	O_2	79	19
**11**	**1**	Cl	CD_3CN	O_2	36	11
**12**	**1**	Cl	CD_3CN/D_2O (9:1)	O_2	38	6
**13**[c]	**1**	OMe	CD_3CN	Ar	22	0
**14**[c]	**1**	OMe	CD_3CN/D_2O (9:1)	Ar	82	0
**15**[c]	**1**	Cl	CD_3CN	Ar	25	0
**16**[c]	**1**	Cl	CD_3CN/D_2O (9:1)	Ar	77	0

[a] Conditions: Catalyst (5 µmol), **2** (100 µmol), solvent (1 mL), 4 × 1.5 W LED 450 nm, 25 °C, oxygen (balloon) or Ar, 8 h [b] Determined by ^1H NMR, [c] electrophotochemistry: constant potential E = 0.3 V, electrolyte $TBAPF_6$ (900 µL, 0.1 M in CH_3CN), working electrode: Pt mesh, counter electrode: Pt wire, pseudo-reference electrode: Ag wire

Table S3. Selective electrophotoxidation of 1-(4-chlorophenyl)ethan-1-ol (**10b**) in presence of thioanisole (**5**) according to **procedure A**

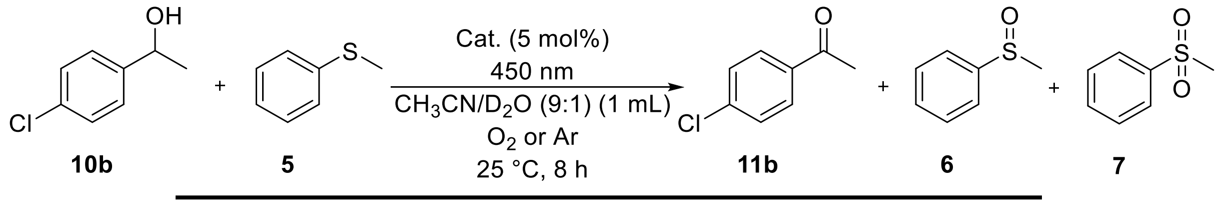

Entry	Catalyst	Atm.	Conversion [%][b]		
			11b	6	7
**1**	**RFTA**	O_2	37	88	12
**2**[c]	**RFTA**	Ar	15	0	0
**3**	**1**	O_2	52	85	15
**4**[c]	**1**	Ar	65	0	0

[a] Conditions: Catalyst (5 µmol), each substrate (50 µmol), solvent (1 mL), 4 × 1.5 W LED 450 nm, 25 °C, oxygen (balloon) or Ar, 8 h, [b] Determined by ^1H NMR, [c] photoelectrochemistry: constant potential E = 0.3 V, electrolyte $TBAPF_6$ (900 µL, 0.1 M in CH_3CN), working electrode: Pt mesh, counter electrode: Pt wire, pseudo-reference electrode: Ag wire

Table S4. Optimization data for the electrophotooxidation of mixture (4-chlorophenyl)methanol (**2b**) and thioanisole (**5**) in presence of various reaction conditions

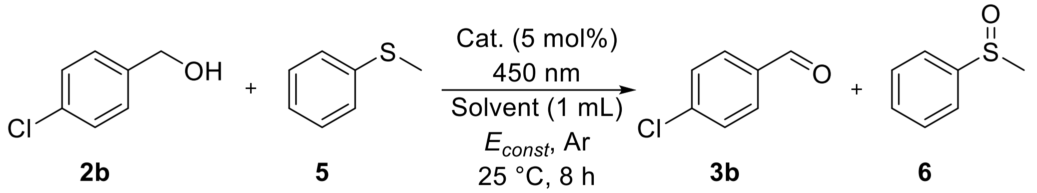

Entry	Catalyst	Solvent	Supporting electrolyte	*E* [V]	Conversion [%][b]	
					**3b**	**6**
**1**	**1**	CD_3CN/D_2O (9:1)	$TBAPF_6$	0.3	64	0
**2**	**1**	CD_3CN/D_2O (9:1)	$TMAPF_6$	0.3	50	0
**3**	**1**	CD_3CN/D_2O (9:1)	$TBABF_4$	0.3	35	0
**4**	**1**	DMF-d_6	$TBAPF_6$	0.3	5	0
**5**	**1**	$CDCl_3$	$TBAPF_6$	0.3	8	0
**6**	**1**	CH_3CN/D_2O (9:1)	$TBAPF_6$	0.3	53	0
**7**	**RFTA**	CH_3CN/D_2O (9:1)	$TBAPF_6$	0.3	14	0
**8**	**8**	CH_3CN/D_2O (9:1)	$TBAPF_6$	0.3	16	0
**9**[c]	**S2**	CH_3CN/D_2O (9:1)	$TBAPF_6$	0.3	7	0
**10**[c]	**S1**	CH_3CN/D_2O (9:1)	$TBAPF_6$	0.3	3	0
**11**	**9**	CH_3CN/D_2O (9:1)	$TBAPF_6$	0.3	64	0
**12**	**1**	CH_3CN/D_2O (9:1)	$TBAPF_6$	0.15	63	0
**13**	**1**	CH_3CN/D_2O (9:1)	$TBAPF_6$	0.45	65	0
**14**	**9**	CH_3CN/D_2O (9:1)	$TBAPF_6$	0.15	73	0
**15**	**9**	CH_3CN/D_2O (9:1)	$TBAPF_6$	0.45	50	0
**16**	-	CH_3CN/D_2O (9:1)	$TBAPF_6$	0.3	0	0
**17**	-	CH_3CN/D_2O (9:1)	$TBAPF_6$	-	0	0
**18**	**1**	CH_3CN/D_2O (9:1)	$TBAPF_6$	-	<5	0
**19**[d]	**1**	CH_3CN/D_2O (9:1)	$TBAPF_6$	0.3	0	0

[a] Conditions: Catalyst (5 µmol), each substrate (50 µmol), solvent (1 mL), 4 × 1.5 W LED 450 nm, 25 °C, Ar, electrolyte $TBAPF_6$ (900 µL, 0.1 M in CH_3CN), working electrode: Pt mesh, counter electrode: Pt wire, pseudo-reference electrode: Ag wire 8 h, [b] Determined by ^1H NMR, [c] irradiated by 400 nm, [d] without irradiation

Table S5. Selectivity study of electrophotoxidation of 1-(p-tolyl)ethan-1-ol (**10c**) according to **procedure A**

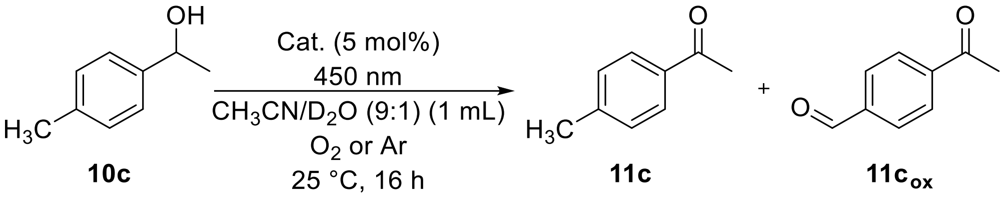

Entry	Catalyst	Atm.	Conversion [%][b]	
			**11c**	**11c$_{ox}$**
**1**	**RFTA**	O_2	70	5
**2**[c]	**RFTA**	Ar	62	0
**3**	**1**	O_2	35	3
**4**[c]	**1**	Ar	70	0

[a] Conditions: Catalyst (5 µmol), **10c** (100 µmol), solvent (1 mL), 4 × 1.5 W LED 450 nm, 25 °C, oxygen (balloon) or Ar, 16 h [b] Determined by ^{1}H NMR, [c] electrophotochemistry: constant potential *E* = 0.3 V, electrolyte $TBAPF_6$ (900 µL, 0.1 M in CH_3CN), working electrode: Pt mesh, counter electrode: Pt wire, pseudo-reference electrode: Ag wire

Table S6. Selectivity study of electrophotoxidation of (4-(4,4,5,5-tetramethyl-1,3,2-dioxaborolan-2-yl)phenyl)methanol (**2h**) according to **procedure A**

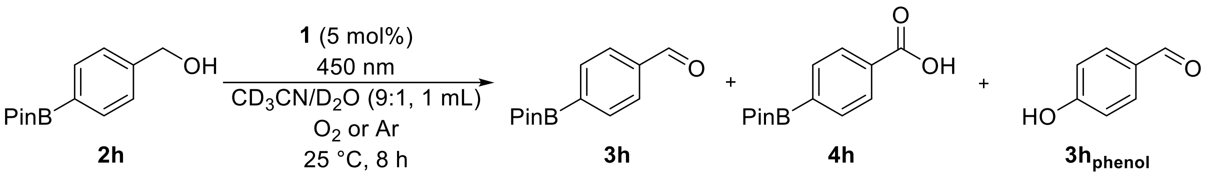

Entry	Atm.	Conversion [%][b]		
		**3h**	**4h**	**3h$_{phenol}$**
**1**	O_2	35	10	35
**2**[c]	Ar	50	0	0

[a] Conditions: Catalyst (5 µmol), **2h** (100 µmol), solvent (1 mL), 4 × 1.5 W LED 450 nm, 25 °C, oxygen (balloon) or Ar, 16 h [b] Determined by ^{1}H NMR, [c] electrophotochemistry: constant potential *E* = 0.3 V, electrolyte $TBAPF_6$ (900 µL, 0.1 M in CH_3CN), working electrode: Pt mesh, counter electrode: Pt wire, pseudo-reference electrode: Ag wire

S3.4 Preparation and characterization of isolated products

4-methoxybenzaldehyde **(3a)**

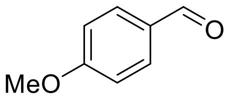

Prepared according to **procedure B** from (4-methoxyphenyl)methanol **2a** (0.138 g, 1.0 mmol, 124 µL). Product **3a** was slightly yellow oil with a yield of 83% (0.113 g). Crude product was purified by flash column chromatography (hexane/EtOAc – 2:1).

^{1}H NMR (400 MHz, CD_3CN) δ 9.86 (s, 1H), 7.85 (d, *J* = 8.7 Hz, 2H), 7.07 (d, *J* = 8.2 Hz, 2H), 3.87 (s, 3H).
^{13}C NMR (101 MHz, CD_3CN) δ 191.9, 165.6, 132.7, 131.0, 115.3, 56.4.
HR-MS (APCI+): calculated for $C_8H_9O_2^+$ ($[M+H]^+$): 137.0597; **found:** 137.0596.

4-chlorobenzaldehyde **(3b)**

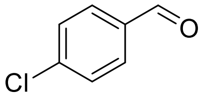

Prepared according to **procedure B** from (4-chlorophenyl)methanol **2b** (0.142 g, 1.0 mmol). Product **3b** was a white solid with yield of 67% (0.095 g), m.p.: 46-47 °C. Crude product was purified by flash column chromatography (hexane/EtOAc – 2:1).

^{1}H NMR (400 MHz, CD_3CN) δ 9.97 (s, 1H), 7.88 (d, *J* = 8.6 Hz, 2H), 7.59 (d, *J* = 8.4 Hz, 2H).
^{13}C NMR (101 MHz, CD_3CN) δ 192.5, 141.0, 136.1, 131.9, 130.3.
HR-MS (APCI+): calculated for $C_7H_6ClO^+$ ($[M+H]^+$): 141.0102; **found:** 141.0099.

4-methylbenzaldehyde **(3c)**

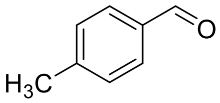

Prepared according to **procedure B** from *p*-tolylmethanol **2c** (0.124 g, 1.0 mmol). Reaction time was 40Sh. Product **3c** was a colourless oil with yield of 69% (0.083 g). Crude product was purified by flash column chromatography (hexane/EtOAc – 2:1).

^{1}H NMR (400 MHz, CD_3CN) δ 9.94 (s, 1H), 7.78 (d, *J* = 8.1 Hz, 2H), 7.39 (d, *J* = 7.9 Hz, 2H), 2.42 (s, 3H).
^{13}C NMR (101 MHz, CD_3CN) δ 193.2, 146.7, 135.4, 130.7, 130.5, 21.8.
HR-MS (APCI+): calculated for $C_8H_9O^+$ ($[M+H]^+$): 121.0648; **found:** 121.0647.

3-chlorobenzaldehyde **(3d)**

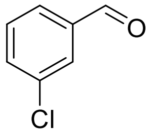

Prepared according to **procedure B** from (3-chlorophenyl)methanol **2d** (0.143 g, 1.0 mmol, 117 µL). Product **3d** was a colourless oil with yield of 33% (0.046 g). Crude product was purified by flash column chromatography (hexane/EtOAc – 2:1).

^{1}H NMR (400 MHz, CD_3CN) δ 9.96 (s, 1H), 7.89 – 7.87 (m, 1H), 7.83 (dt, *J* = 7.6, 1.3 Hz, 1H), 7.68 (dd, *J* = 8.0, 2.2 Hz, 1H), 7.57 (t, *J* = 7.8 Hz, 1H).
^{13}C NMR (101 MHz, CD_3CN) δ 192.4, 139.1, 135.7, 135.1, 131.8, 129.7, 129.1.
HR-MS (APCI+): calculated for $C_7H_6ClO^+$ ($[M+H]^+$): 141.0102; **found:** 141.0098.

2-chlorobenzaldehyde **(3e)**

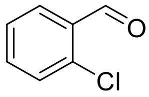

Prepared according to **procedure B** from (2-chlorophenyl)methanol **2e** (0.144 g, 1.0 mmol). Product **3e** was a white solid with yield of 20% (0.028 g), m.p.: 57-59 °C. Crude product was purified by flash column chromatography (hexane/EtOAc – 2:1).

^{1}H NMR (400 MHz, CD_3CN) δ 10.42 (s, 1H), 7.88 (dd, *J* = 7.7, 1.8 Hz, 1H), 7.62 (ddd, *J* = 8.1, 7.2, 1.8 Hz, 1H), 7.54 (dd, *J* = 8.1, 1.2 Hz, 1H), 7.47 (ddd, *J* = 7.9, 7.3, 1.2 Hz, 1H).
^{13}C NMR (101 MHz, CD_3CN) δ 190.7, 138.1, 136.4, 133.4, 131.7, 130.3, 128.6.
HR-MS (APCI+): calculated for $C_7H_6ClO^+$ ($[M+H]^+$): 141.0102; **found:** 141.0099.

4-(trifluoromethyl)benzaldehyde **(3f)**

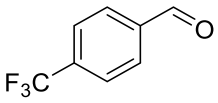

Prepared according to **procedure B** from (4-(trifluoromethyl)phenyl)methanol **2f** (0.176 g, 1.0 mmol, 137 µL). Product **3f** was a colourless oil with yield of 13% (0.022 g). Crude product was purified by flash column chromatography (hexane/EtOAc – 2:1).

^{1}H NMR (400 MHz, CD_3CN) δ 10.09 (s, 1H), 8.06 (d, *J* = 7.9 Hz, 2H), 7.88 (d, *J* = 8.2 Hz, 2H).

^{13}C NMR (101 MHz, CD_3CN) δ 192.9, 140.1, 135.5 (q, J_F = 32.3 Hz), 130.9, 127.1 (q, J_F = 4.0 Hz), 124.8 (q, J_F = 272.0 Hz).

^{19}F NMR (376 MHz, CD_3CN) δ -63.34.

HR-MS (APCI+): calculated for $C_8H_6F_3O^+$ ($[M+H]^+$): 175.0365; **found:** 175.0363.

4-(methylthio)benzaldehyde **(3g)**

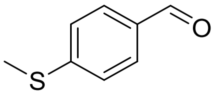

Prepared according to **procedure B** from (4-(methylthio)phenyl)methanol **2g** (0.154 g, 1.0 mmol). Reaction time was 48h. Product **3g** was a yellowish oil with a yield of 7% (0.010 g). Crude product was purified by flash column chromatography (hexane/EtOAc – 3:1).

^{1}H NMR (400 MHz, CD_3CN) δ 9.90 (s, 1H), 7.79 (d, *J* = 6.5 Hz, 2H), 7.41 (d, *J* = 6.5 Hz, 2H), 2.54 (s, 3H).

^{13}C NMR (101 MHz, CD_3CN) δ 192.5, 148.8, 134.1, 130.7, 126.1, 14.7.

HR-MS (APCI+): calculated for $C_8H_9OS^+$ ($[M+H]^+$): 153.0369; **found:** 153.0366.

4-(4, 4, 5, 5-tetramethyl-1, 3, 2-dioxaborolan-2-yl)benzaldehyde **(3h)**

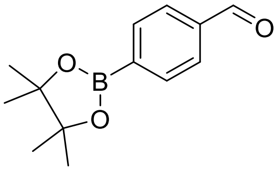

Prepared according to **procedure B** from (4-(4, 4, 5, 5-tetramethyl-1, 3, 2-dioxaborolan-2-yl)phenyl)methanol **2h** (0.234 g, 1.0 mmol). Product **3h** was a white solid with a yield of 46% (0.106 g), m.p.: 58-59 °C. Crude product was purified by flash column chromatography (hexane/EtOAc – 4:1).

^{1}H NMR (400 MHz, CD_3CN) δ 10.03 (s, 1H), 7.89 (s, 4H), 1.34 (s, 12H).

^{13}C NMR (101 MHz, CD_3CN) δ 193.9, 139.4, 135.9, 129.6, 85.3, 30.9, 25.2.

^{11}B NMR (101 MHz, CD_3CN) δ 29.55 (s).

HR-MS (APCI+): calculated for $C_{13}H_{18}BO_3^+$ ($[M+H]^+$): 233.1344; **found:** 233.1353.

methyl 4-formylbenzoate **(3i)**

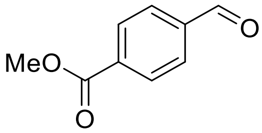

Prepared according to **procedure B** from methyl 4-(hydroxymethyl)benzoate **2i** (0.234 g, 1.0 mmol). Reaction time was 48h. Product **3i** was a white solid with a yield of 40% (0.065 g), m.p.: 58-60°C. Crude product was purified by flash column chromatography (hexane/EtOAc – 4:1).

^{1}H NMR (400 MHz, CD_3CN) δ 10.08 (s, 1H), 8.15 (d, *J* = 7.7 Hz, 2H), 7.98 (d, *J* = 6.9 Hz, 2H), 3.90 (s, 3H).

^{13}C NMR (101 MHz, CD_3CN) δ 193.3, 166.9, 140.4, 136.0, 130.9, 130.4, 53.2.

HR-MS (APCI+): calculated for $C_{10}H_{11}O_3^+$ ($[M+CH_3]^+$): 179.0703; **found:** 179.0700.

1-(4-methoxyphenyl)ethan-1-one **(11a)**

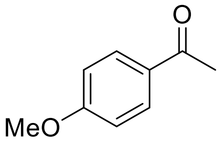

Prepared according to **procedure B** from 1-(4-methoxyphenyl)ethan-1-ol **10a** (0.152 g, 1.0 mmol, 141 µL). Reaction time was 40h. Product **11a** was a colourless oil with yield of 99% (0.149 g). Crude product was purified by flash column chromatography (hexane/EtOAc – 2:1).

^{1}H NMR (400 MHz, CD_3CN) δ 7.93 (d, *J* = 8.9 Hz, 2H), 6.99 (d, *J* = 8.9 Hz, 2H), 3.85 (s, 3H), 2.50 (s, 3H).

^{13}C NMR (101 MHz, CD_3CN) δ 197.4, 164.4, 131.4, 131.3, 114.6, 56.3, 26.7.

HR-MS (APCI+): calculated for $C_9H_{11}O_2^+$ ($[M+H]^+$): 151.0754; **found:** 151.0751.

1-(4-chlorophenyl)ethan-1-one **(11b)**

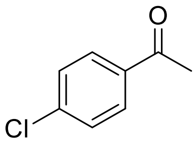

Prepared according to **procedure B** from 1-(4-chlorophenyl)ethan-1-ol **10b** (0.157 g, 1.0 mmol, 134 µL). Product **11b** was a colourless oil with yield of 74% (0.115 g). Crude product was purified by flash column chromatography (hexane/EtOAc – 2:1).

^{1}H NMR (400 MHz, CD_3CN) δ 7.93 (d, *J* = 8.7 Hz, 2H), 7.51 (d, *J* = 8.6 Hz, 2H), 2.55 (s, 3H).
^{13}C NMR (101 MHz, CD_3CN) δ 197. 9, 139.7, 136.8, 130.9, 129.7, 27.0.
HR-MS (APCI-): calculated for $C_8H_6ClO^-$ ($[M\text{-}H]^-$): 153.0113; **found:** 153.0108.

1-(p-tolyl)ethan-1-one **(11c)**

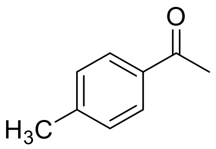

Prepared according to **procedure B** from 1-(p-tolyl)ethan-1-ol **10c** (0.136 g, 1.0 mmol, 138 µL). Product **11c** was a colourless oil with yield of 36% (0.049 g). Crude product was purified by flash column chromatography (hexane/EtOAc – 2:1).

^{1}H NMR (400 MHz, CD_3CN) δ 7.86 (d, *J* = 8.3 Hz, 2H), 7.31 (d, *J* = 7.9 Hz, 2H), 2.53 (s, 3H), 2.39 (s, 3H).
^{13}C NMR (101 MHz, CD_3CN) δ 198.5, 144.9, 135.8, 130.2, 129.3, 26.9, 21.6.
HR-MS (APCI+): calculated for $C_9H_{11}O^+$ ($[M+H]^+$): 135.0804; **found:** 135.0807.

1-(4-methylthiophenyl)ethan-1-one **(11d)**

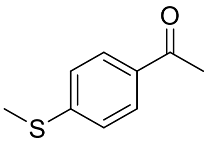

Prepared according to **procedure B** from 1-(4-(methylthio)phenyl)ethan-1-ol **10d** (0.165 g, 1.0 mmol). Reaction time was 48 h. Product **11d** was a white solid with yield of 20% (0.034 g), m.p.: 74-76 °C. Crude product was purified by flash column chromatography (hexane/EtOAc – 3:1).

^{1}H NMR (400 MHz, CD_3CN) δ 7.88 (d, *J* = 8.8 Hz, 2H), 7.33 (d, *J* = 8.6 Hz, 2H), 2.53 (s, 3H), 2.52 (s, 3H).
^{13}C NMR (101 MHz, CD_3CN) δ 198.0, 146.8, 134.5, 129.6, 125.7, 26.8, 14.8.
HR-MS (APCI+): calculated for $C_9H_{11}OS^+$ ($[M+H]^+$): 167.0525; **found:** 167.0527.

1-(4-methoxyphenyl)propan-1-one **(11e)**

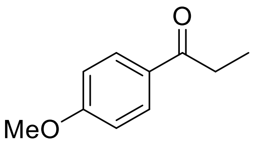

Prepared according to **procedure B** from 1-(4-methoxyphenyl)propan-1-ol **10e** (0.166 g, 1.0 mmol, 153 µL). Reaction time was 24 h. Product **11e** was a yellowish oil with a yield of 53% (0.087 g). Crude product was purified by flash column chromatography (hexane/EtOAc – 4:1).

^{1}H NMR (400 MHz, CD_3CN) δ 7.93 (d, *J* = 5.8 Hz, 2H), 6.97 (d, *J* = 5.8 Hz, 2H), 3.84 (s, 3H), 2.94 (q, *J* = 7.0 Hz, 2H), 1.11 (t, *J* = 7.4 Hz, 3H).
^{13}C NMR (101 MHz, CD_3CN) δ 200.0, 164.3, 131.0, 114.6, 56.2, 32.0, 8.6.
HR-MS (APCI+): calculated for $C_{10}H_{13}O_2^+$ ($[M+H]^+$): 165.0910; **found**: 165.0908.

S3.5 NMR spectra of isolated products

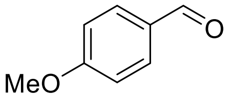

3a

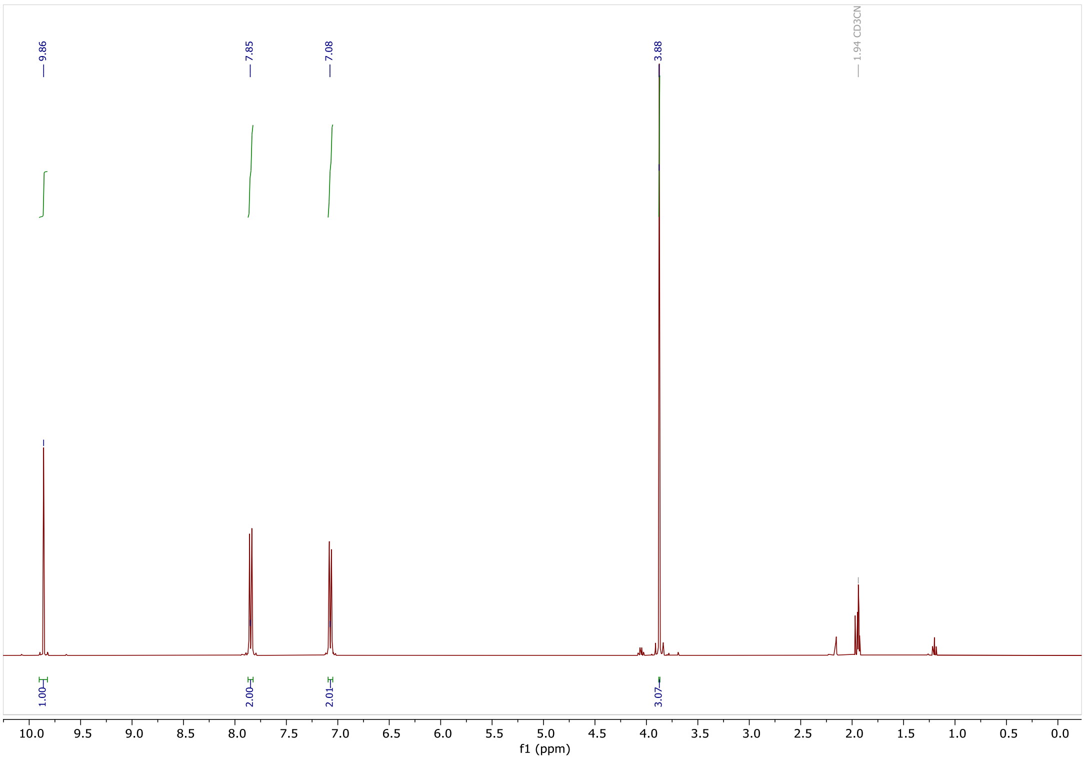

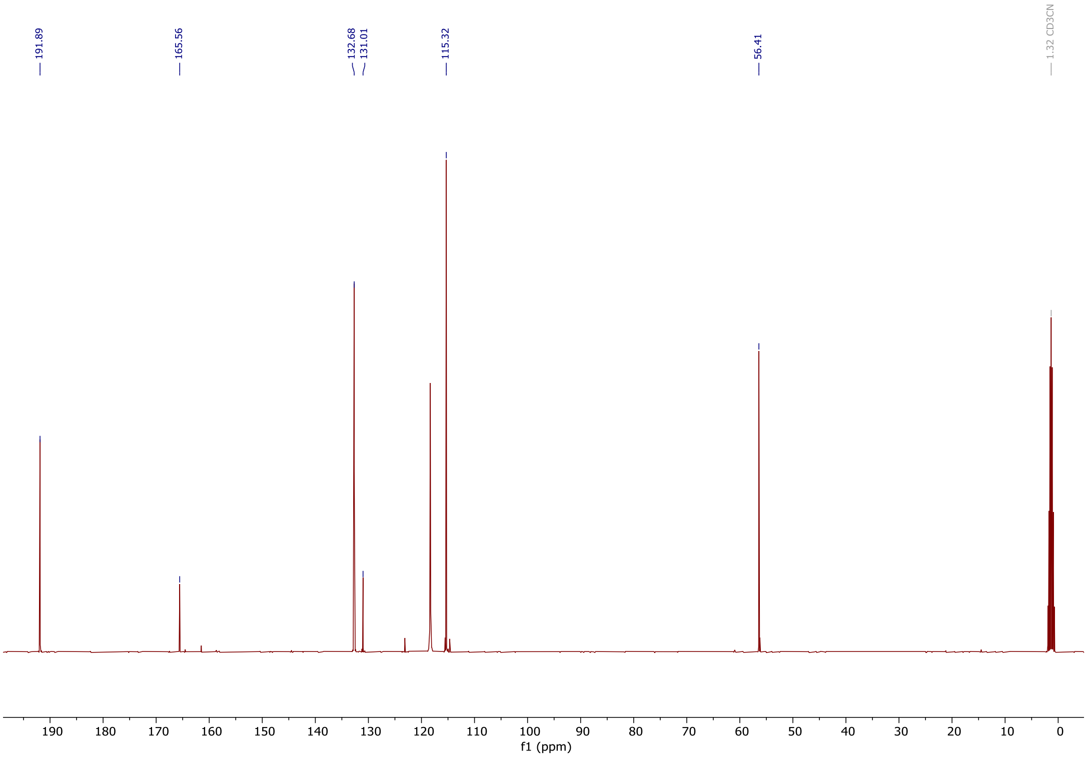

Figure S2. ^{1}H (400 MHz) and ^{13}C (101 MHz) NMR spectra of **3a** in CD_3CN.

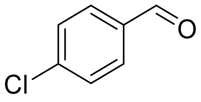

3b

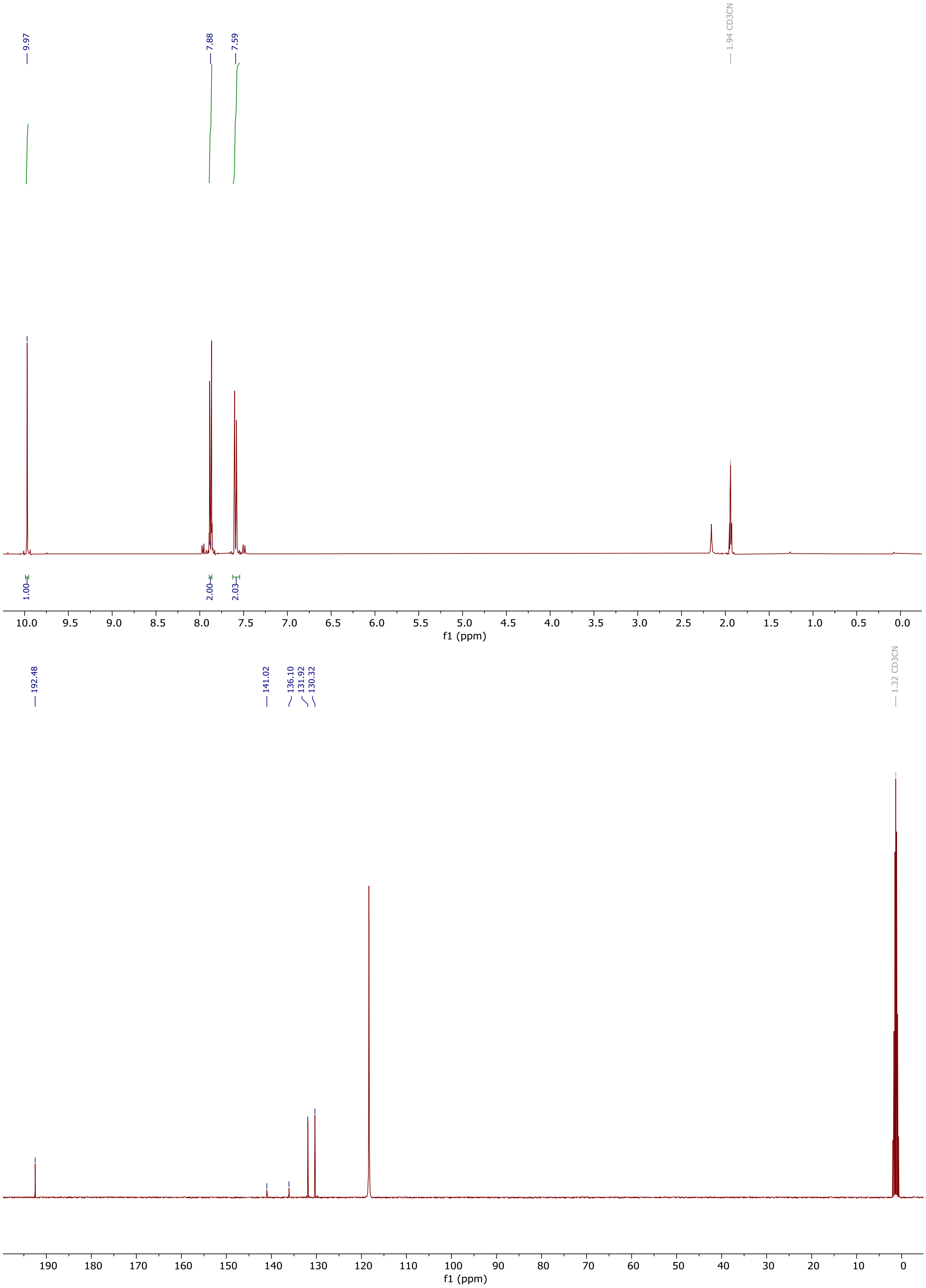

Figure S3. ^{1}H (400 MHz) and ^{13}C (101 MHz) NMR spectra of **3b** in CD_3CN.

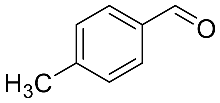

3c

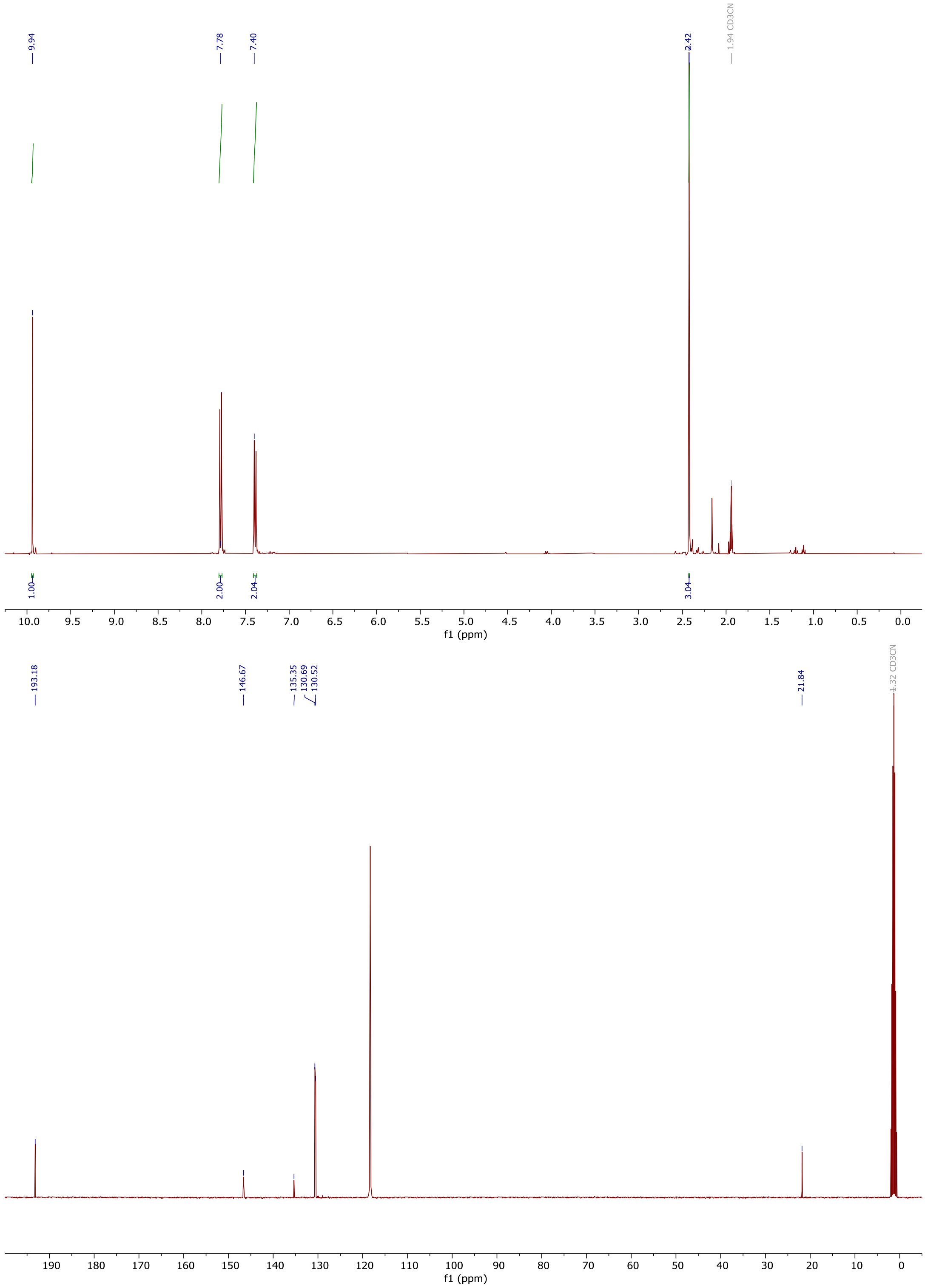

Figure S4. ^{1}H (400 MHz) and ^{13}C (101 MHz) NMR spectra of **3c** in CD_3CN.

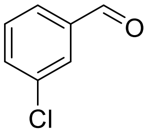

3d

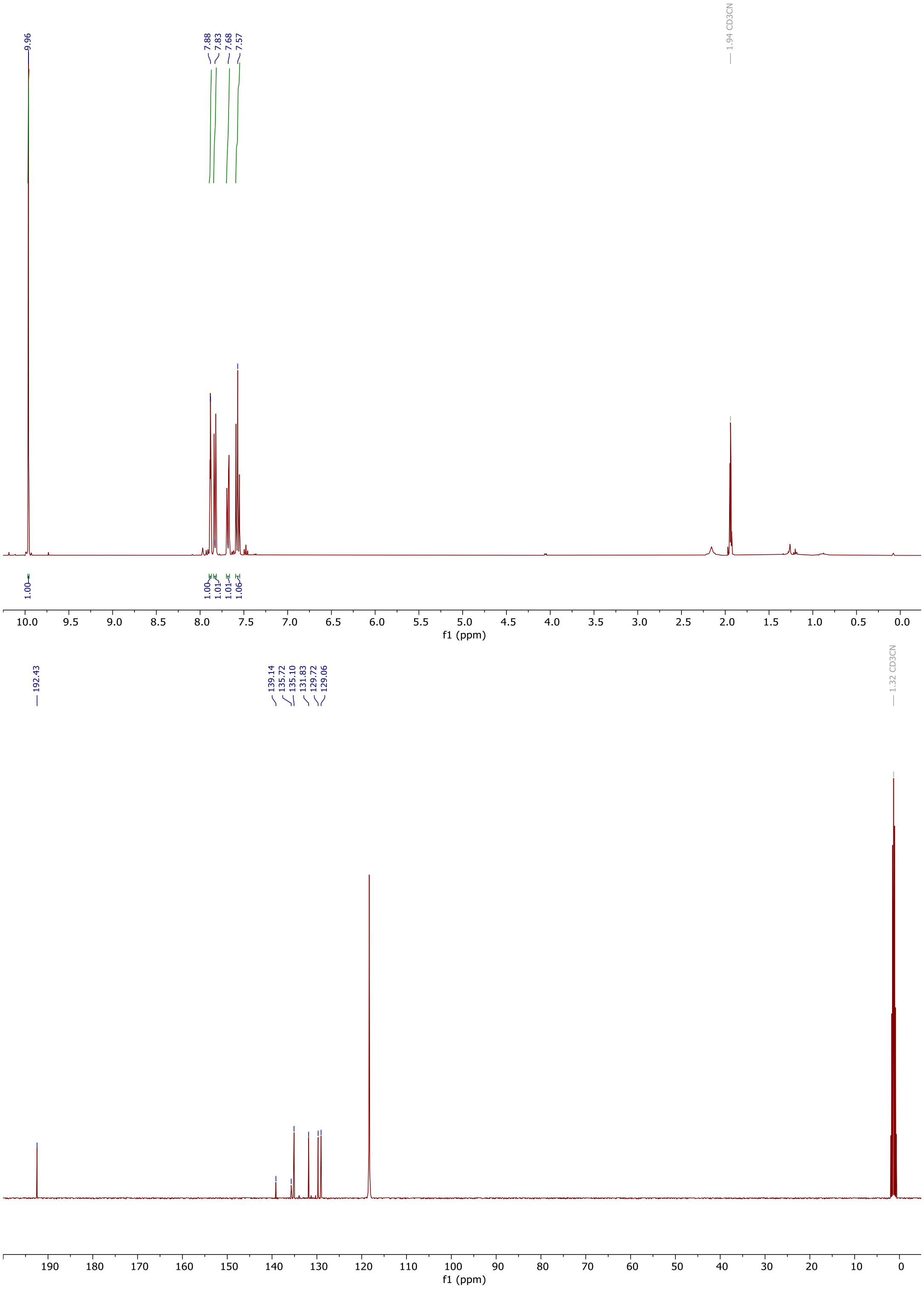

Figure S5. ^{1}H (400 MHz) and ^{13}C (101 MHz) NMR spectra of **3d** in CD_3CN.

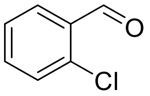

3e

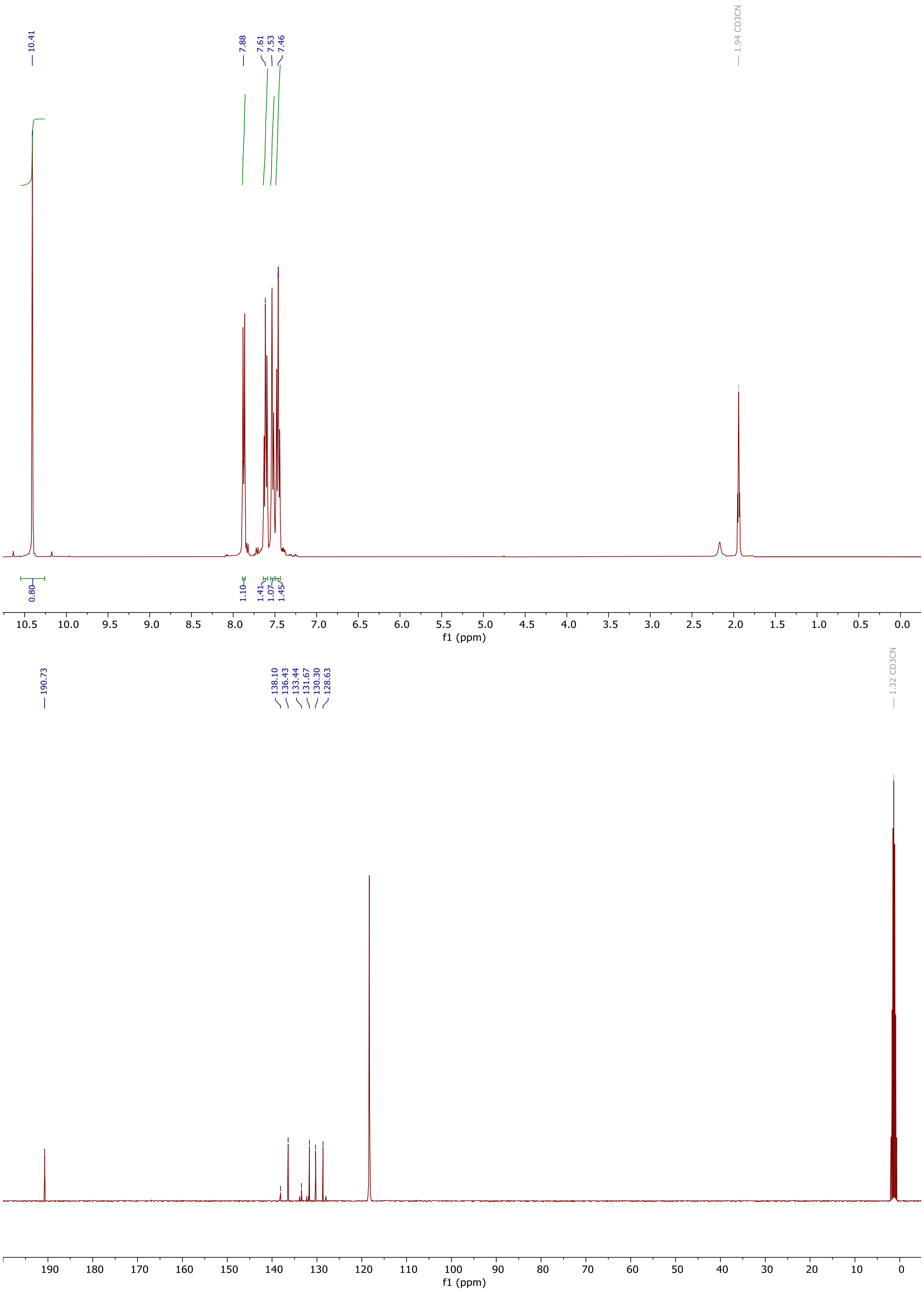

Figure S6. ^{1}H (400 MHz) and ^{13}C (101 MHz) NMR spectra of **3e** in CD_3CN.

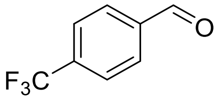
F3C
O

3f

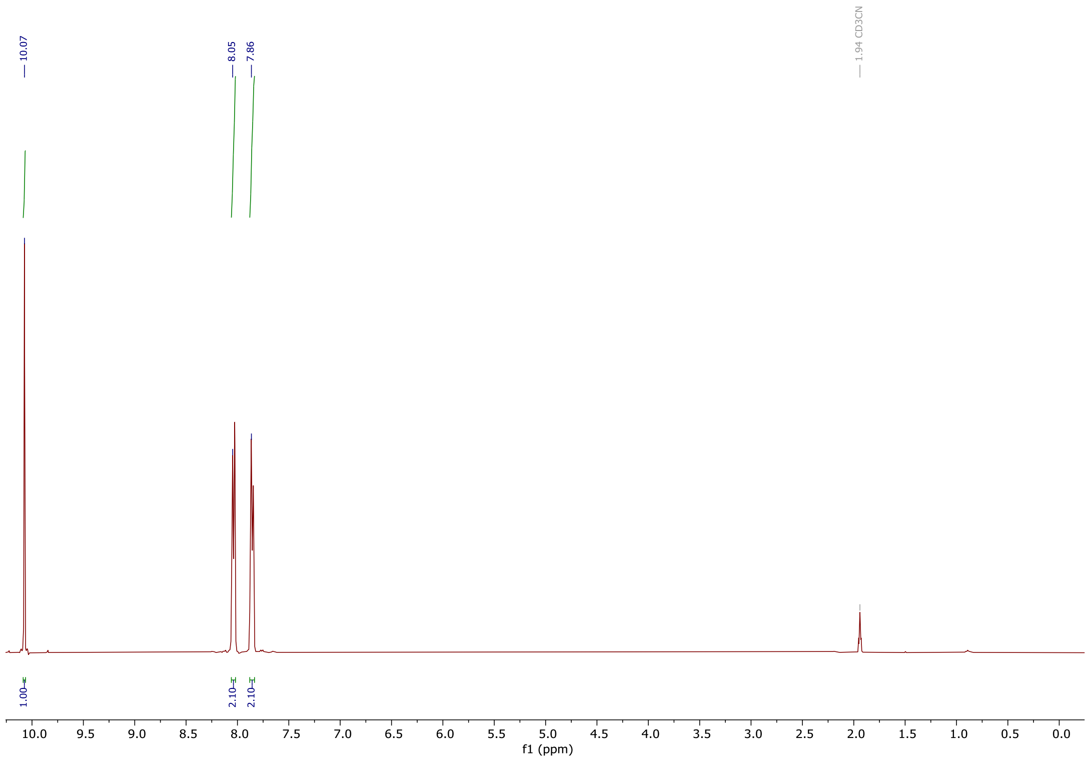
10.07
8.05
7.86
1.94 CD3CN
1.00
2.10
2.10
f1 (ppm)

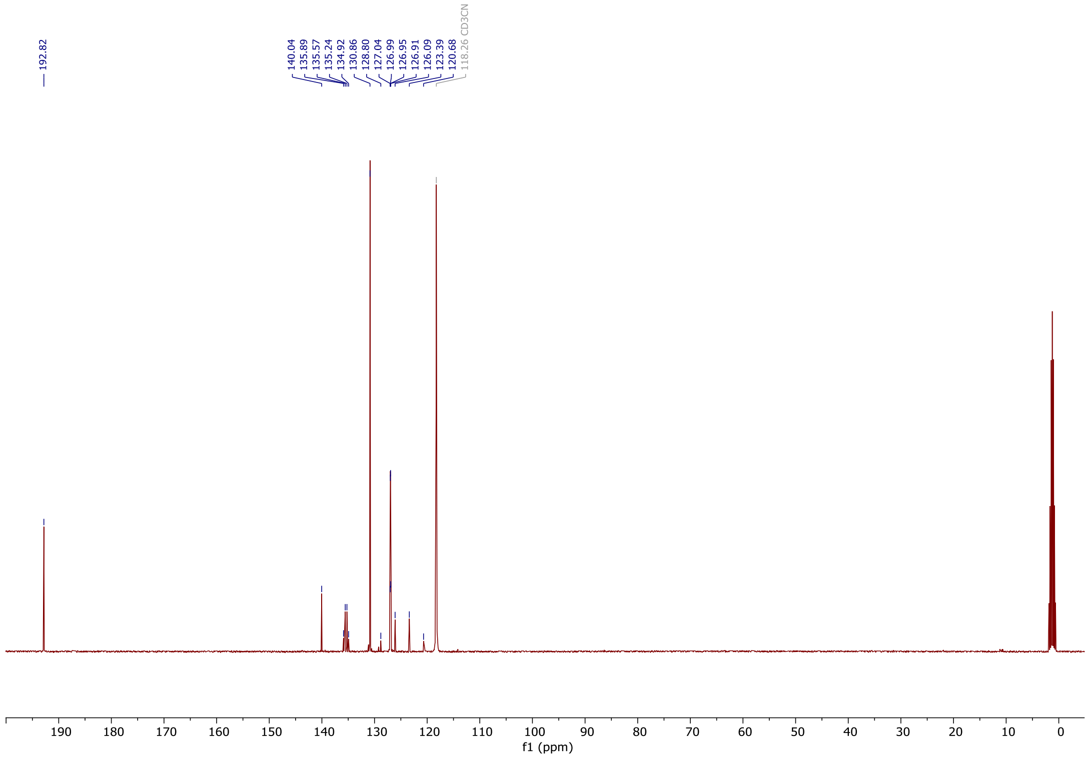
192.82
140.04
135.89
135.57
135.24
134.92
130.86
128.80
127.04
126.99
126.95
126.91
126.09
123.39
120.68
118.26 CD3CN
f1 (ppm)

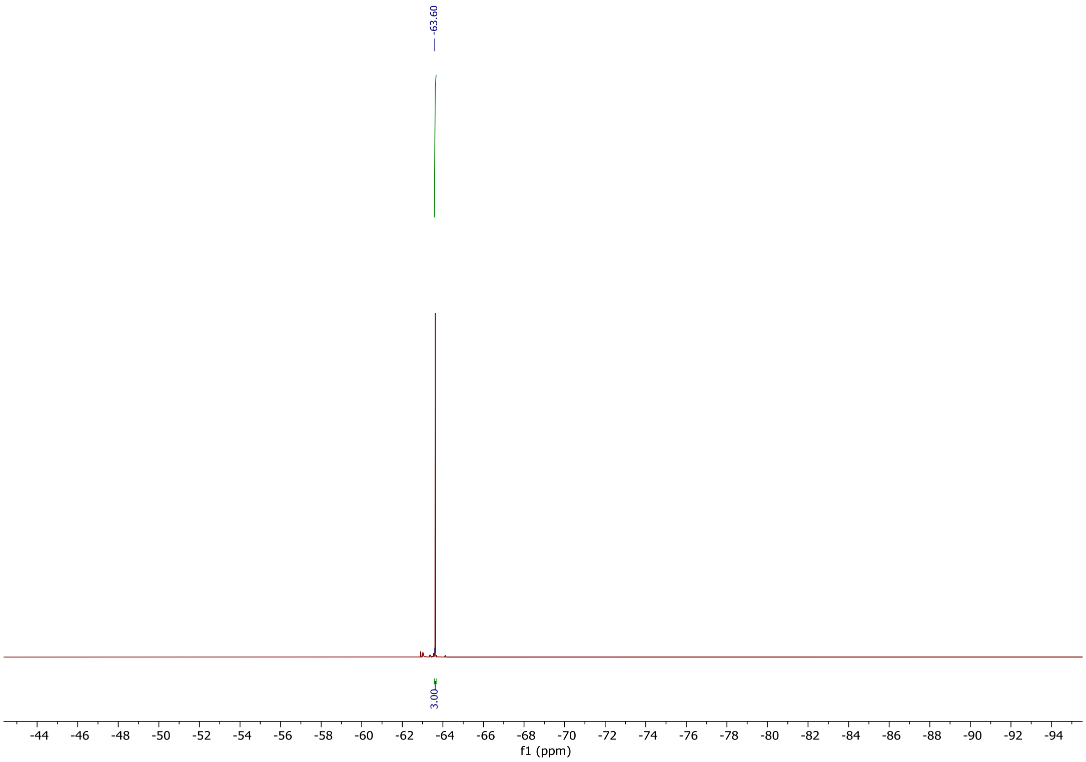

Figure S7. ^{1}H (400 MHz), ^{13}C (101 MHz), and ^{19}F (376 MHz) NMR spectra of **3f** in CD_3CN.

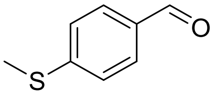

3g

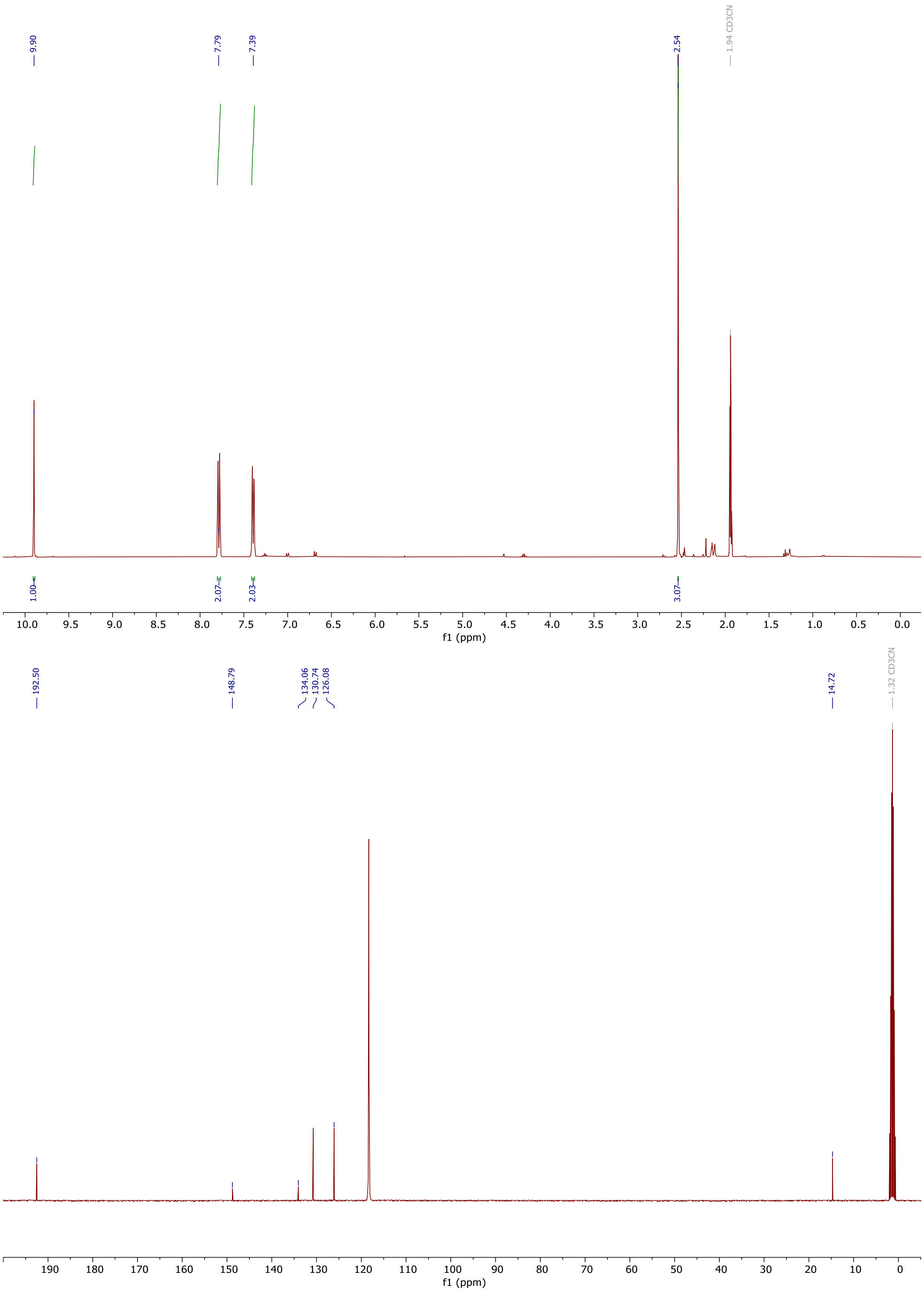

Figure S8. ^{1}H (400 MHz) and ^{13}C (101 MHz) NMR spectra of **3g** in CD_3CN.

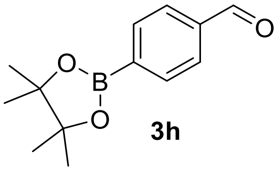
O
B
O
O

3h

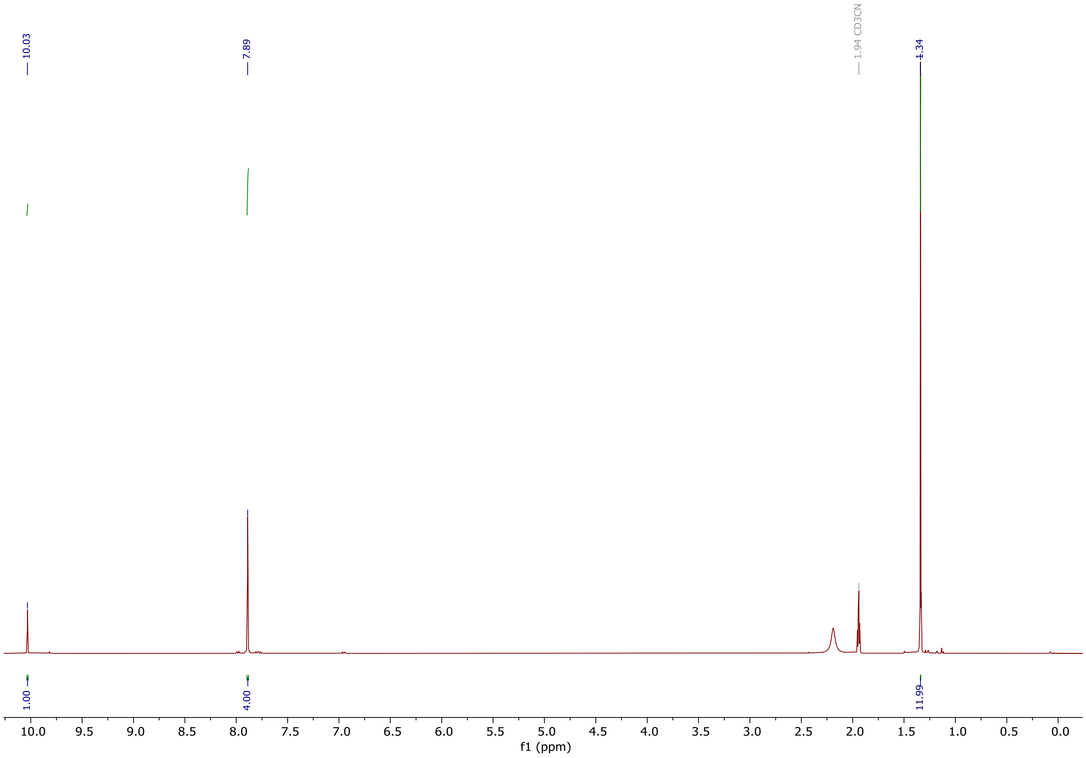
10.03
7.89
1.94 CD3CN
1.34
1.00
4.00
11.99
10.0 9.5 9.0 8.5 8.0 7.5 7.0 6.5 6.0 5.5 5.0 4.5 4.0 3.5 3.0 2.5 2.0 1.5 1.0 0.5 0.0
f1 (ppm)

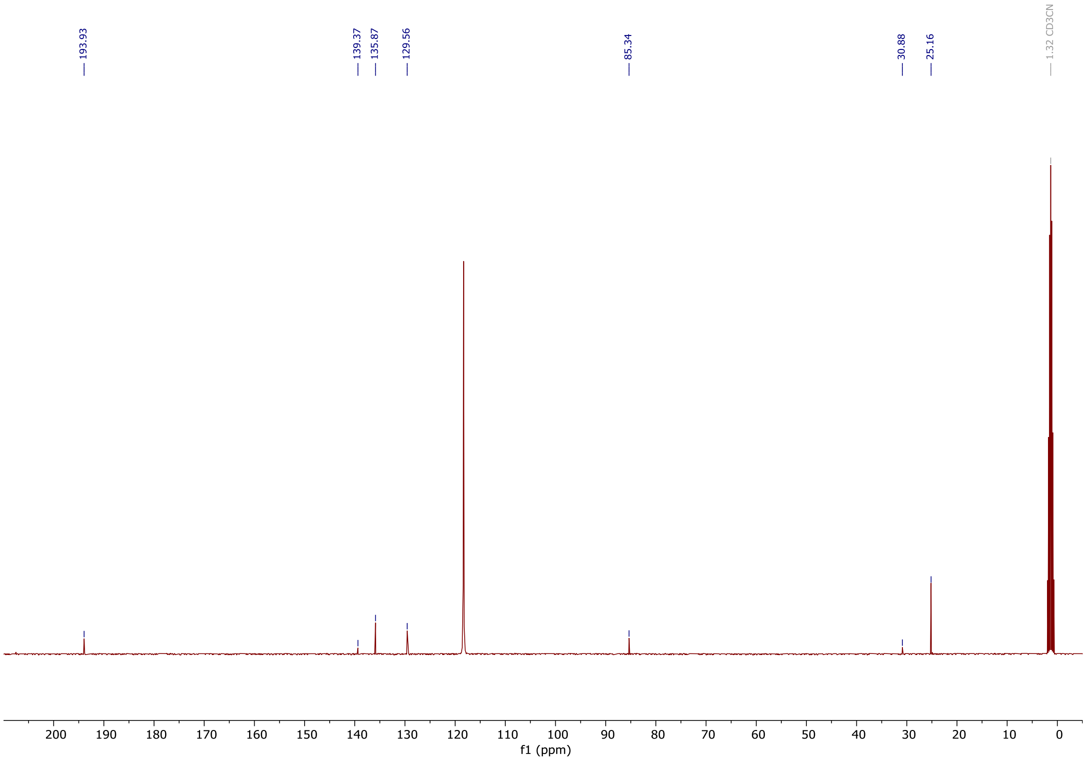
193.93
139.37
135.87
129.56
85.34
30.88
25.16
1.32 CD3CN
200 190 180 170 160 150 140 130 120 110 100 90 80 70 60 50 40 30 20 10 0
f1 (ppm)

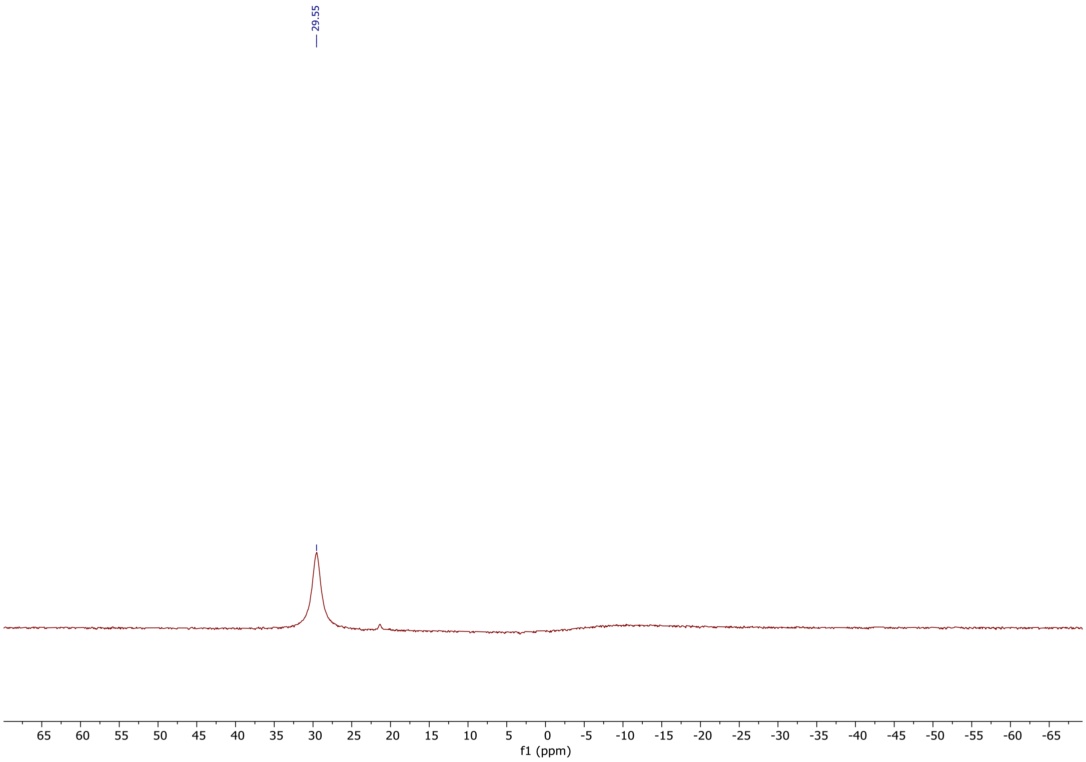

Figure S9. ^{1}H (400 MHz), ^{13}C (101 MHz), and ^{11}B (101 MHz) NMR spectra of **3h** in CD_3CN.

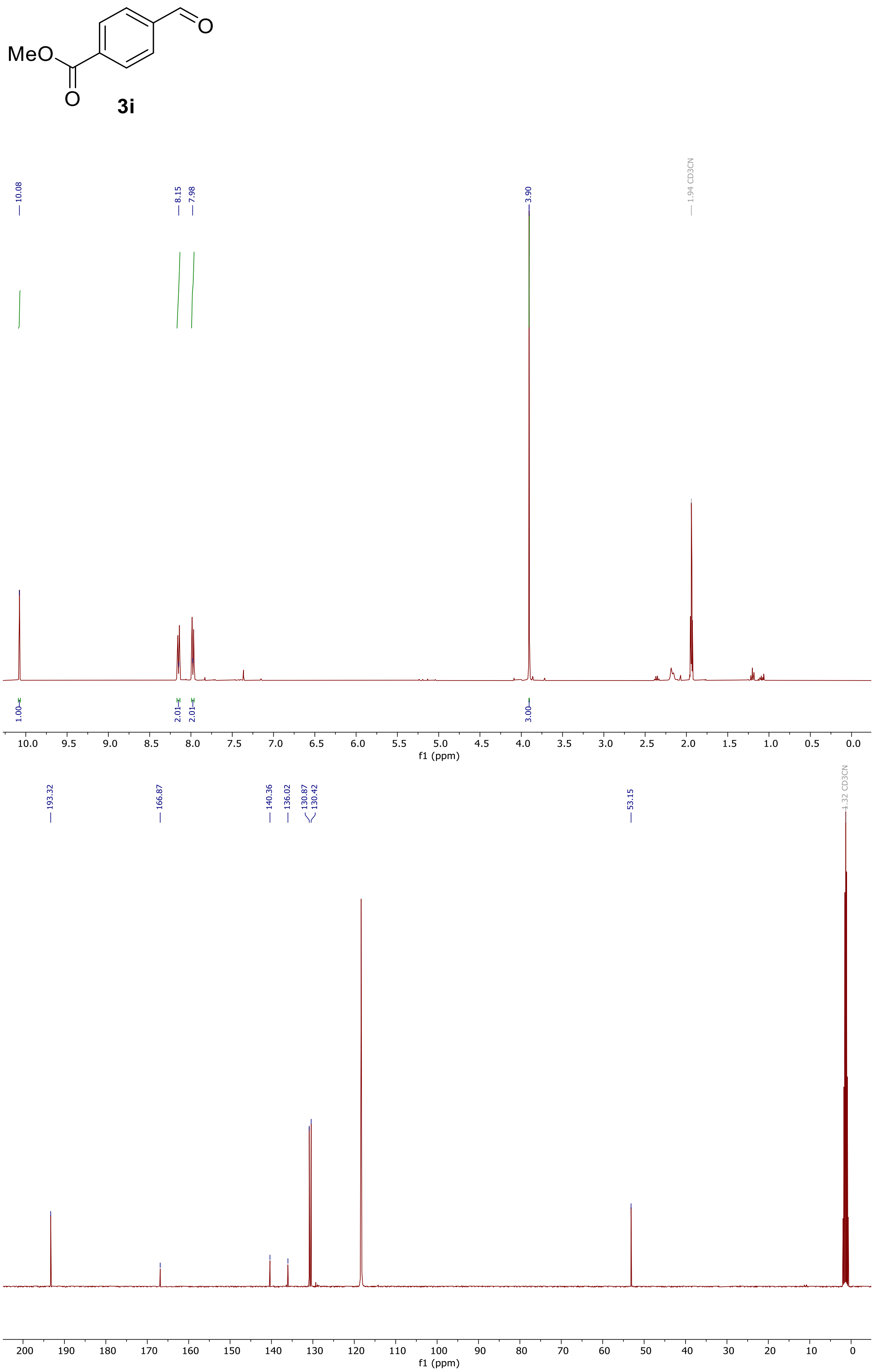

Figure S10. ^{1}H (400 MHz) and ^{13}C (101 MHz) NMR spectra of **3i** in CD_3CN.

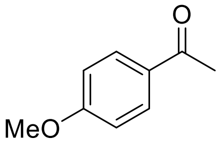

11a

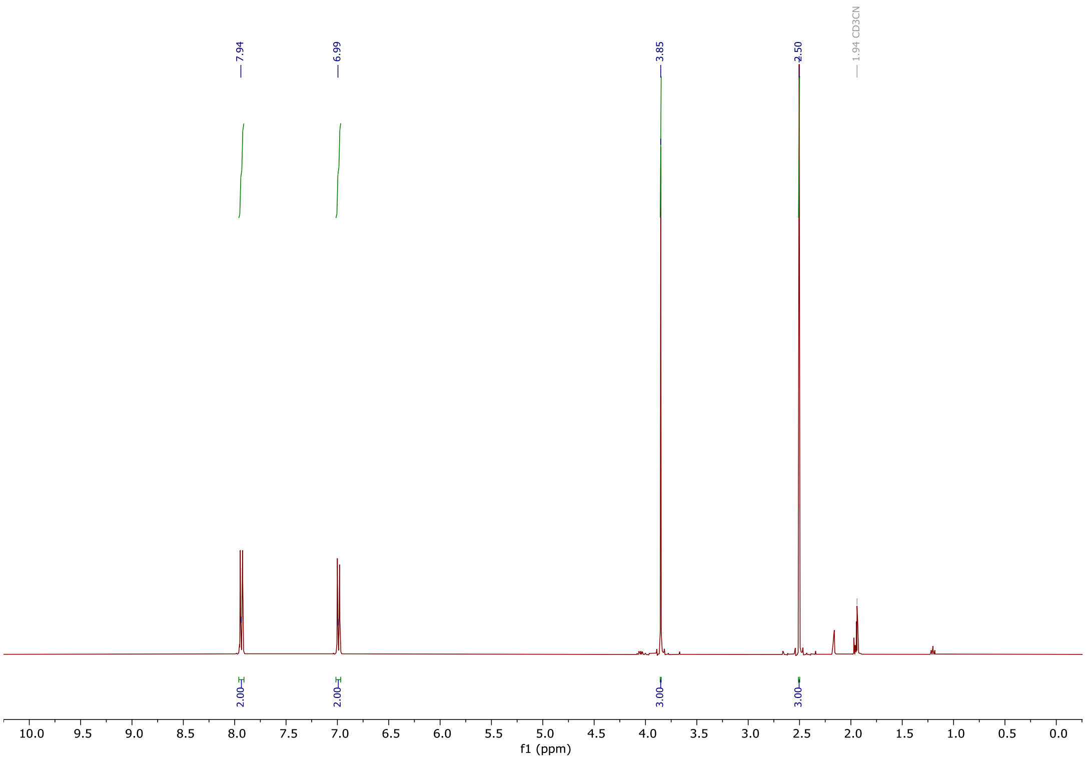

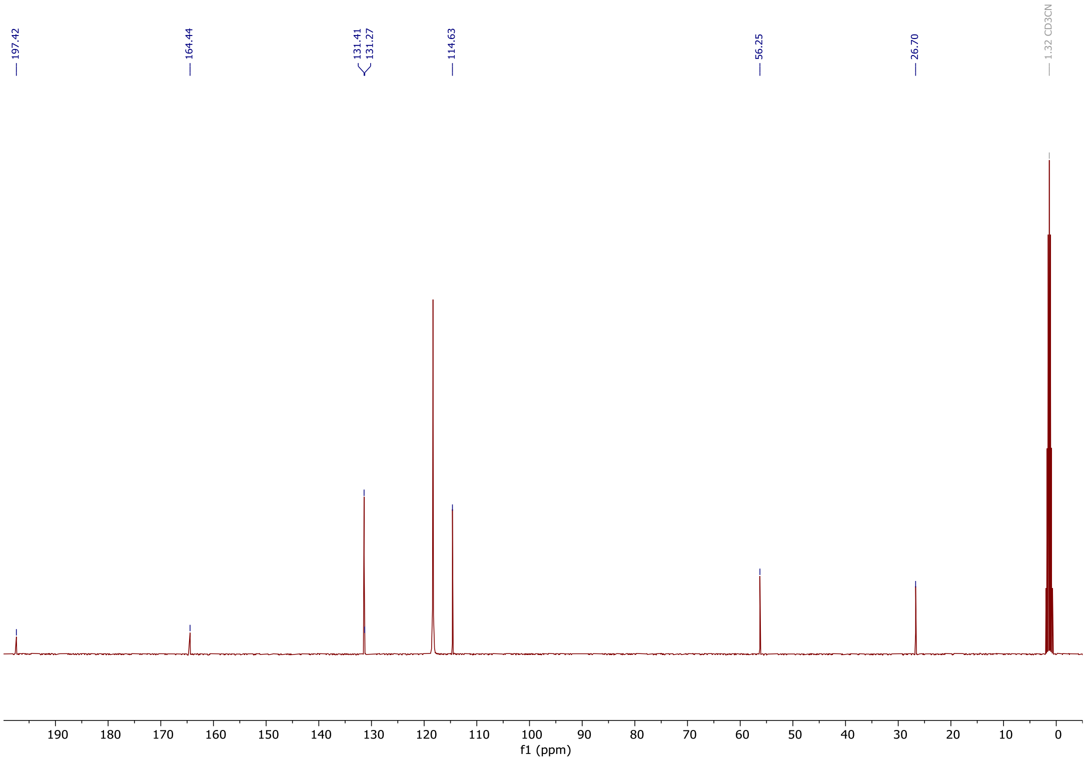

Figure S11. ^{1}H (400 MHz) and ^{13}C (101 MHz) NMR spectra of **11a** in CD_3CN.

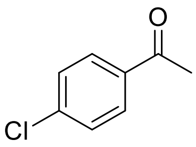

11b

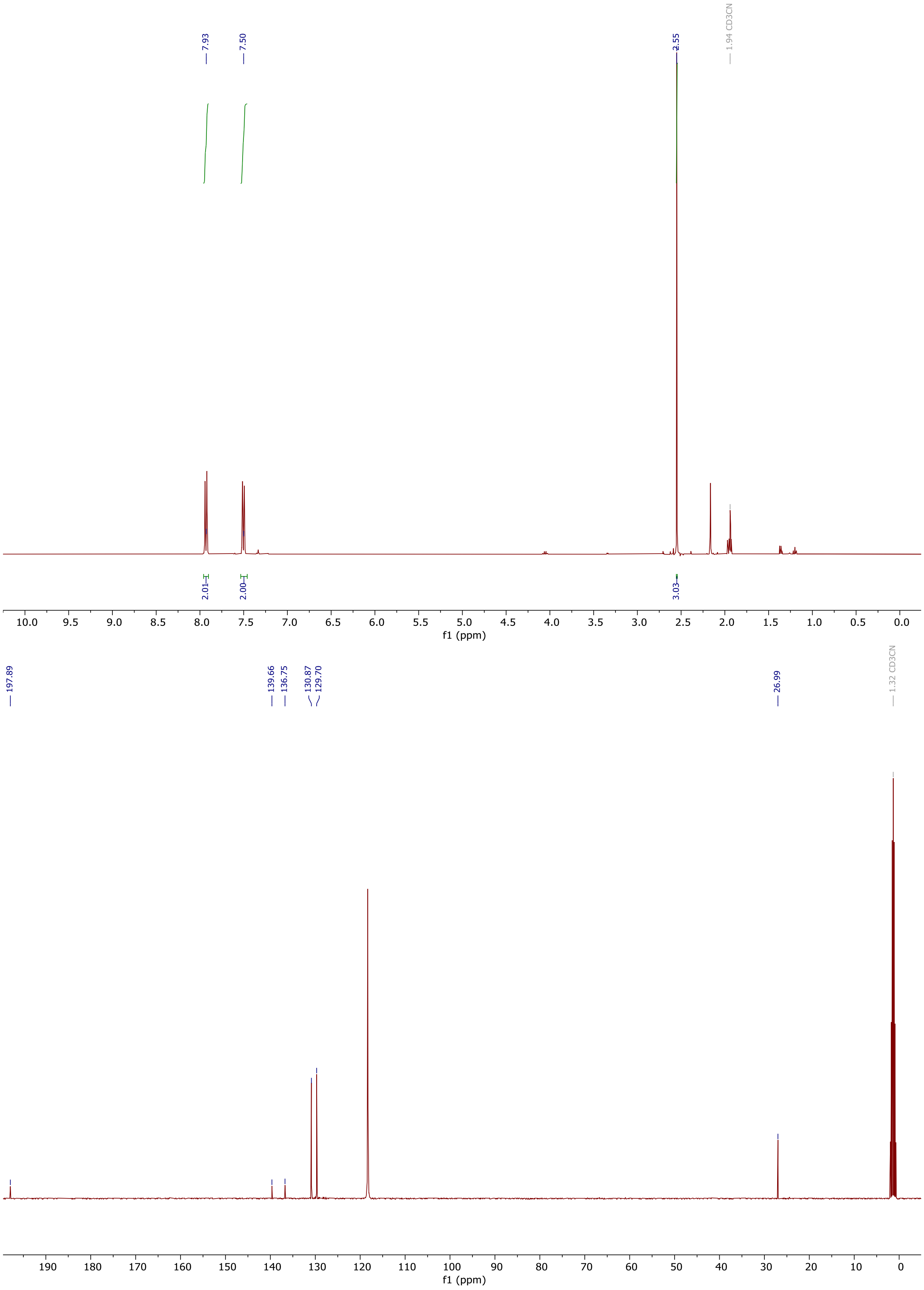

Figure S12. ^{1}H (400 MHz) and ^{13}C (101 MHz) NMR spectra of **11b** in CD_3CN.

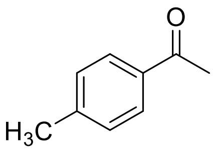

11c

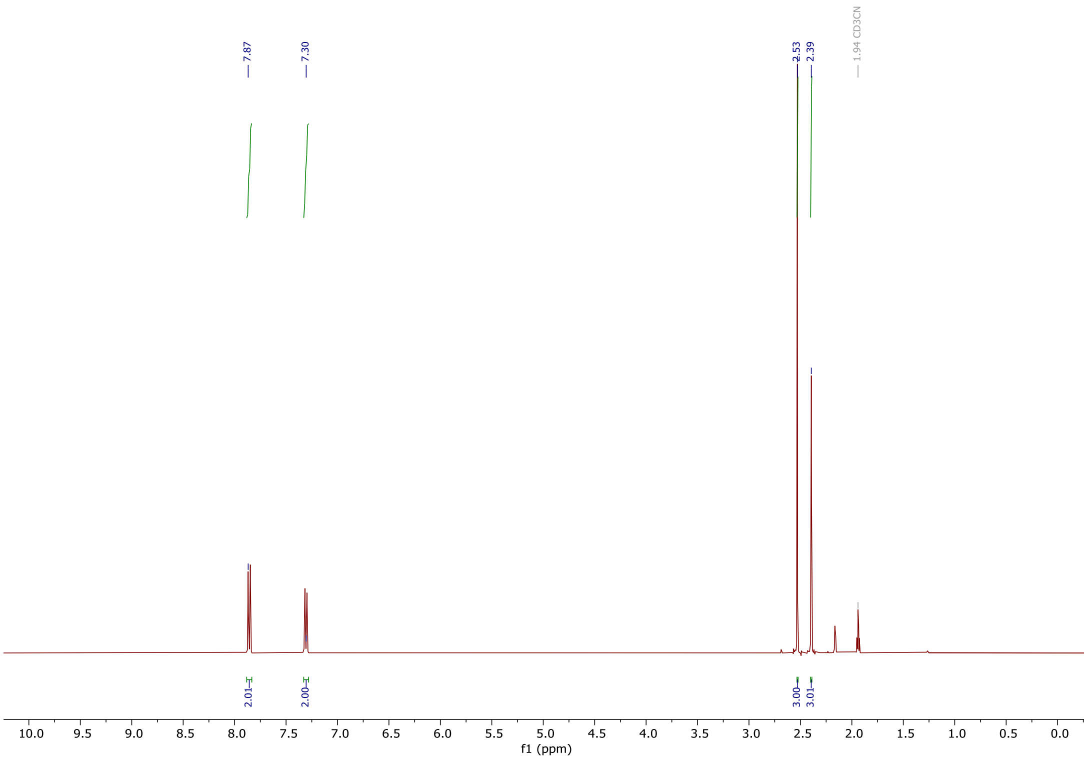

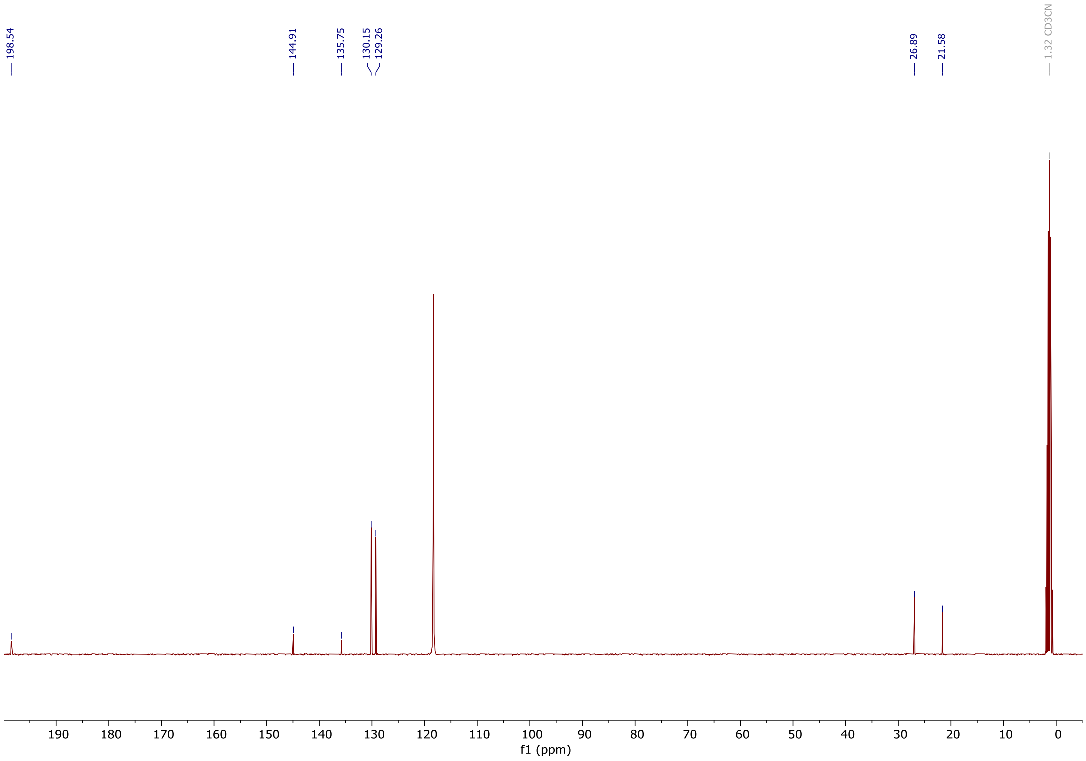

Figure S13. ^{1}H (400 MHz) and ^{13}C (101 MHz) NMR spectra of **11c** in CD_3CN.

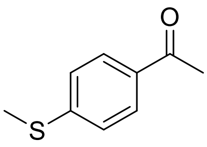

11d

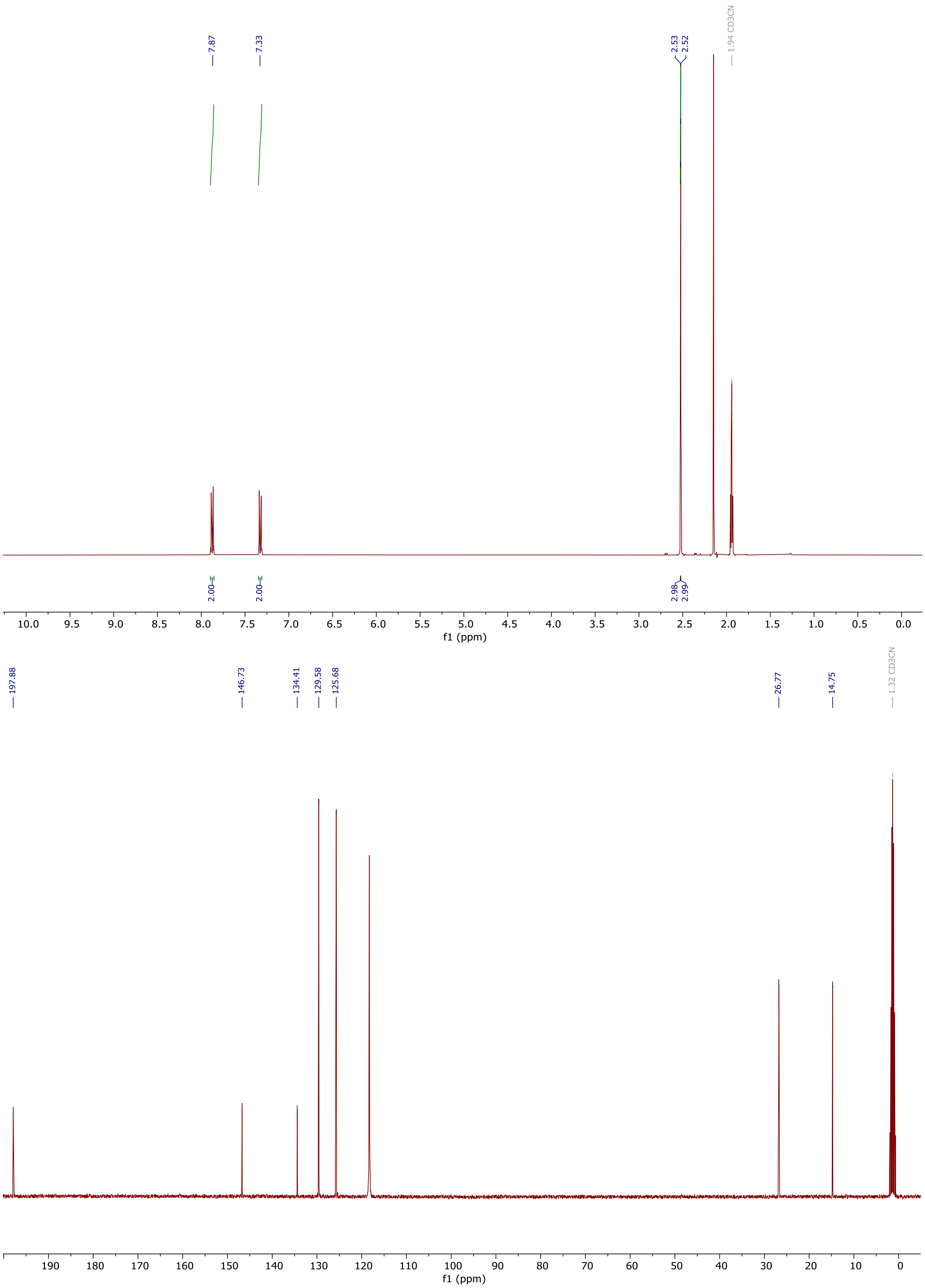

Figure S14. ^{1}H (400 MHz) and ^{13}C (101 MHz) NMR spectra of **11d** in CD_3CN.

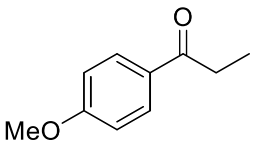

11e

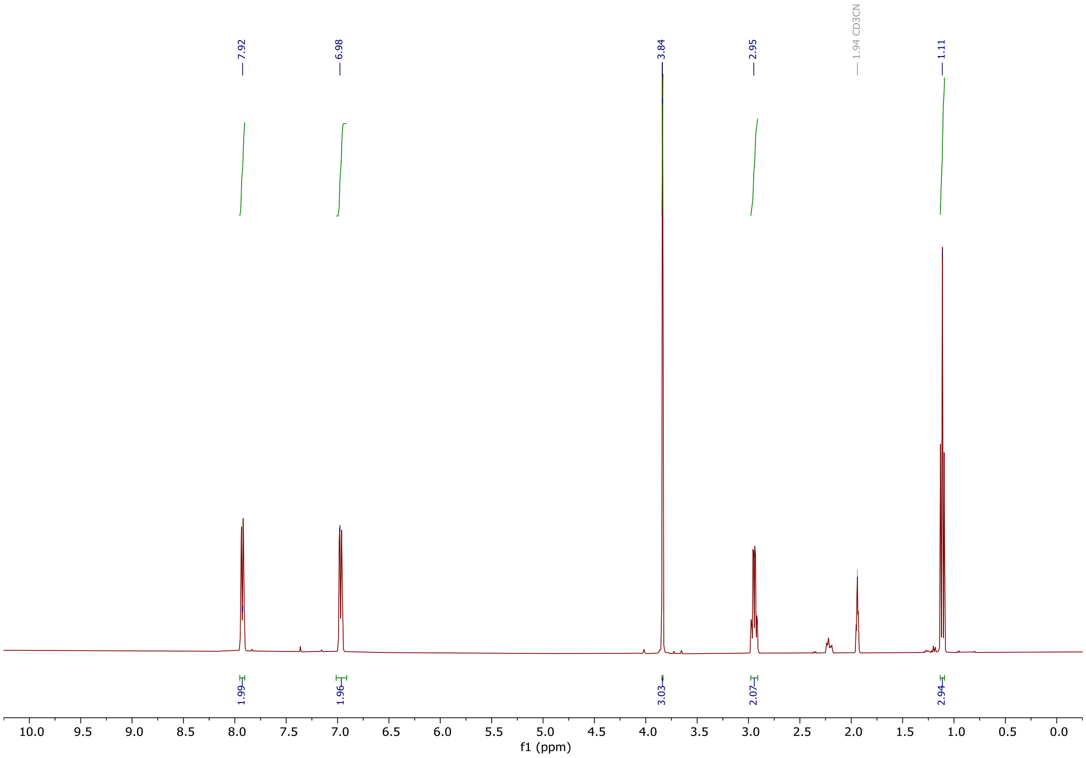

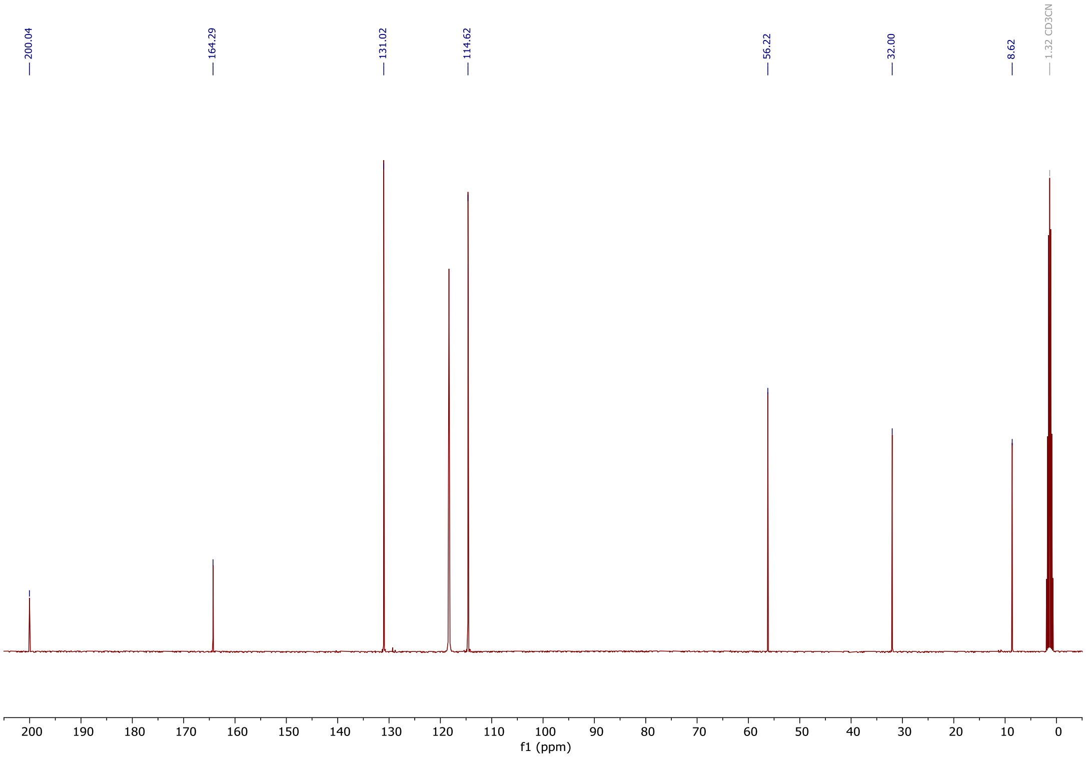

Figure S15. ^{1}H (400 MHz) and ^{13}C (101 MHz) NMR spectra of **11e** in CD_3CN.

S3.6 Comparison of NMR spectra of experiments conducted under O_2 and Ar atmosphere

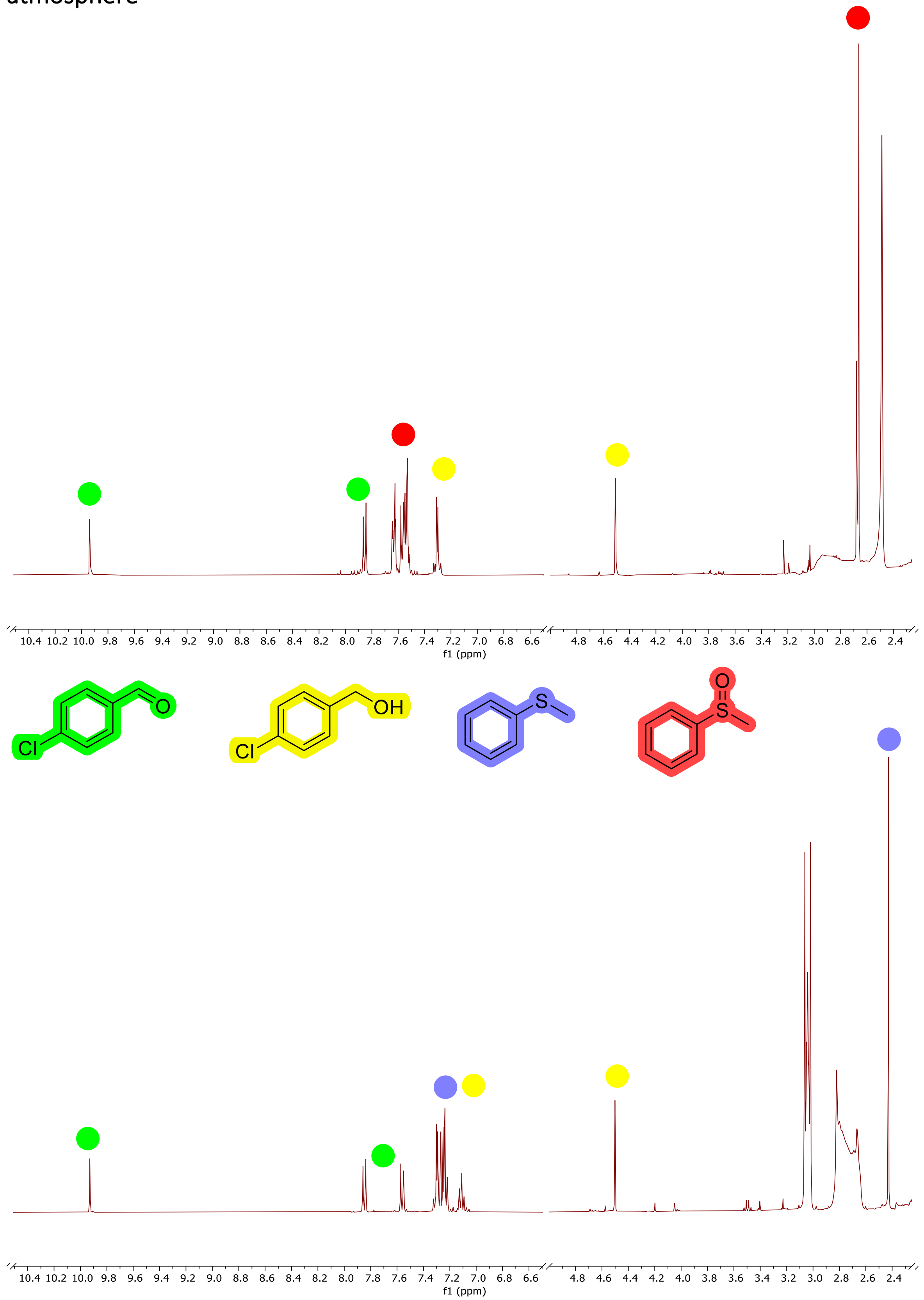

Figure S16. Comparison of ^{1}H NMR spectra of crude mixtures of experiments conducted under O_2 (up) and under electrophotochemical conditions (down) in CD_3CN.

S4. Spectroelectrochemical data

S4.1 Electrochemical redox potential of catalysts

Table S7. Half-wave potentials of chosen catalysts. Potentials are related to SCE.

Catalyst	$E_{1/2}$ [V]
**RFTA**	-0.81[3]
**1a**	-0.41[10]
**8**	-0.64[10]
**9**	-0.17
**S1**	-1.33[10]
**S2**	-1.45[10]

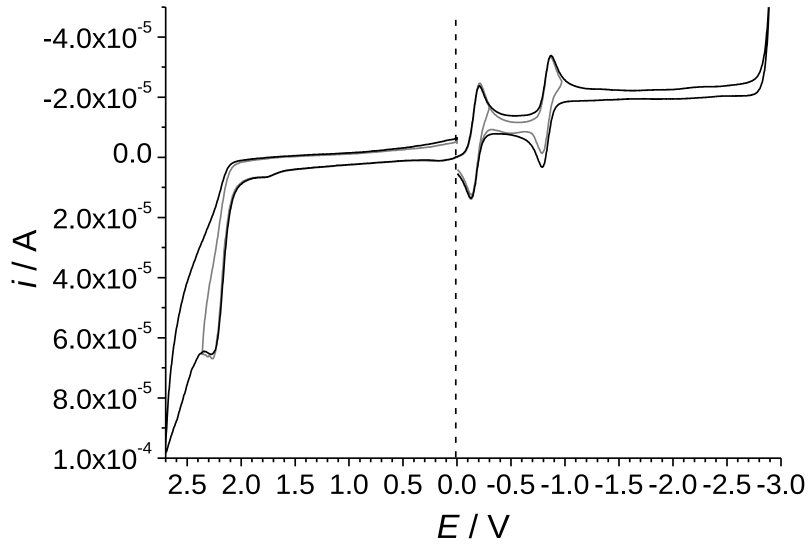

Figure S17. Cyclic voltammetry in polarographic convention of flavinium salt **9** in CH_3CN using Glassy carbon working electrode (3 mm GC disk RDE electrode); counter electrode: Platinum sheet (ca. 1 cm^2); pseudo reference electrode: Silver wire (Metrohm, anhydrous), scan rate 100 $mV{\cdot}s^{-1}$, concentration of flavin $c = 1 \times 10^{-3}$ M, 0.1 M Me_4NPF_6, vs. SCE. Initial potential 0 V, switching potentials 2.55 V and -3.0 V

S4.2 UV stability of photocatalyst in the mixture with substrates

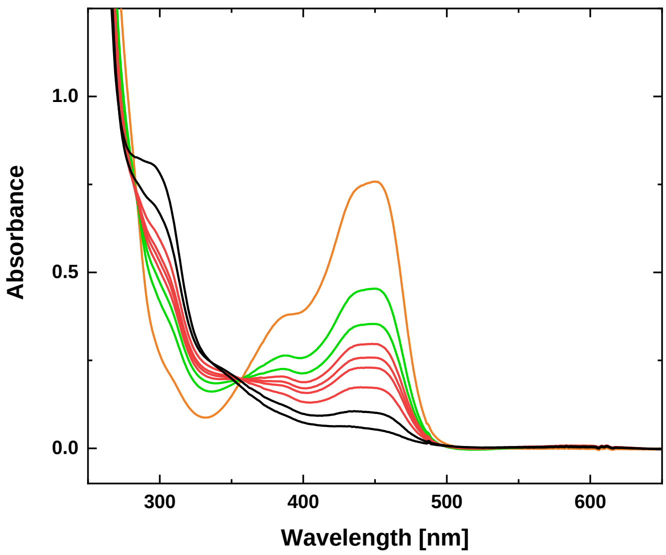

Figure S18. Stability of flavinium salt **1a** (c = 3.3 × 10^{-5} mol/dm^3) in the presence of 1-(4-(methylthio)phenyl)ethan-1-ol (**10d**) (c = 1.0 × 10^{-4} mol/dm^3) under Ar atmosphere and irradiation (λ = 460 nm, t = 10 min).

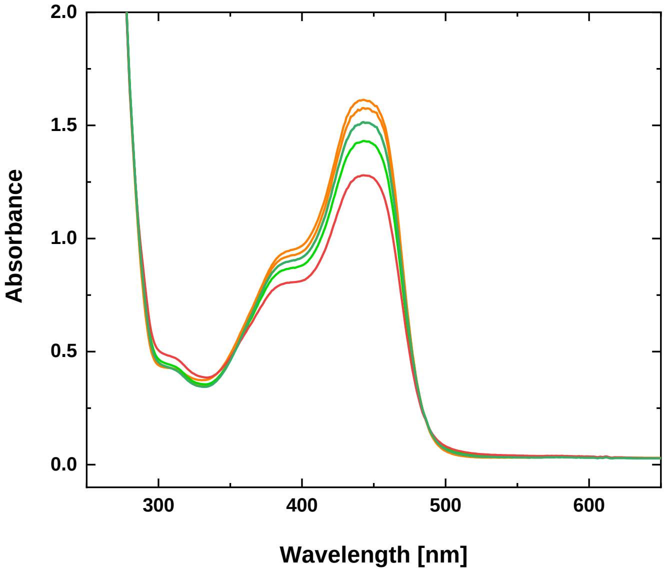

Figure S19. Stability of flavinium salt **1a** (c = 1.0 × 10^{-4} mol/dm^3) in the presence of (4-chlorophenyl)methanol (**2b**) (c = 1.0 × 10^{-4} mol/dm^3) under Ar atmosphere and irradiation (λ = 460 nm, t = 10 min).

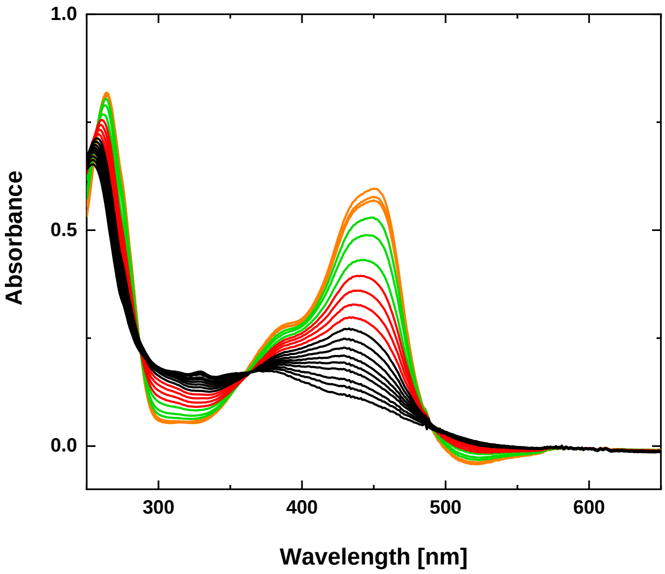

Figure S20. Stability of flavinium salt **1a** (*c* = 3.3 × 10^{-5} mol/dm^3) in the presence of (4-chlorophenyl)methanol (**2b**) (*c* = 1.0 × 10^{-4} mol/dm^3) under Ar atmosphere and irradiation (λ = 460 nm, *t* = 120 min).

S4.3 Electrochemical spectra

The dependence of current and charge to time was measured during each electrophotocatalytic oxidation. Reactions were controlled based on the current progress and reaction was prolonged if neccessary.

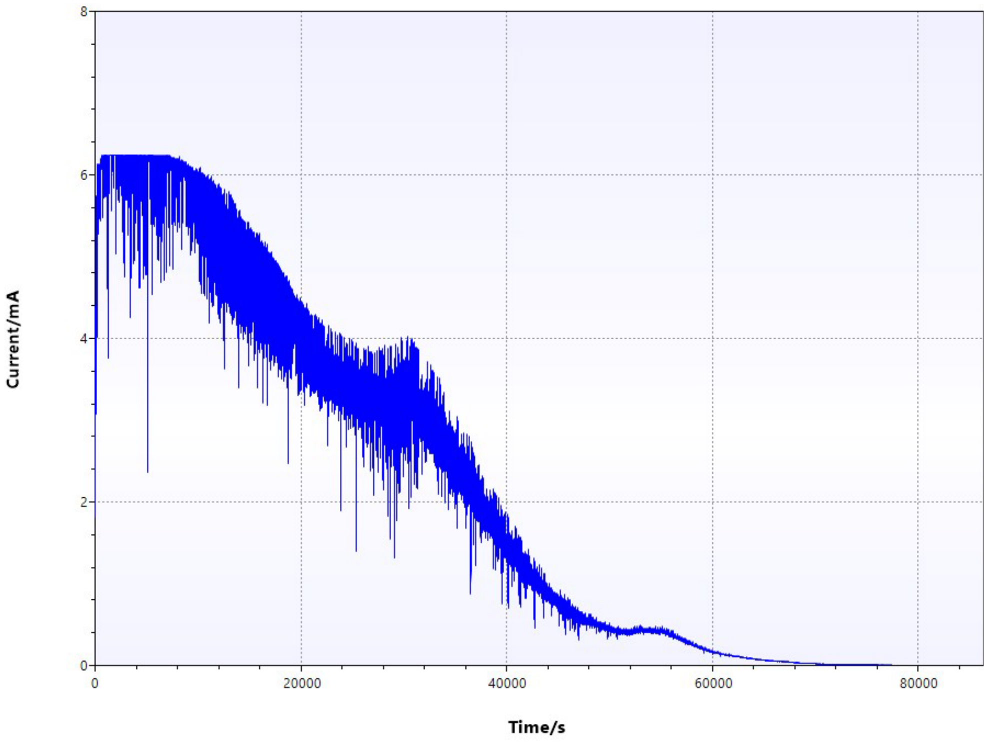

Figure S21. Current-time relationship for oxidation of (4-methoxyphenyl)methanol (**2a**)

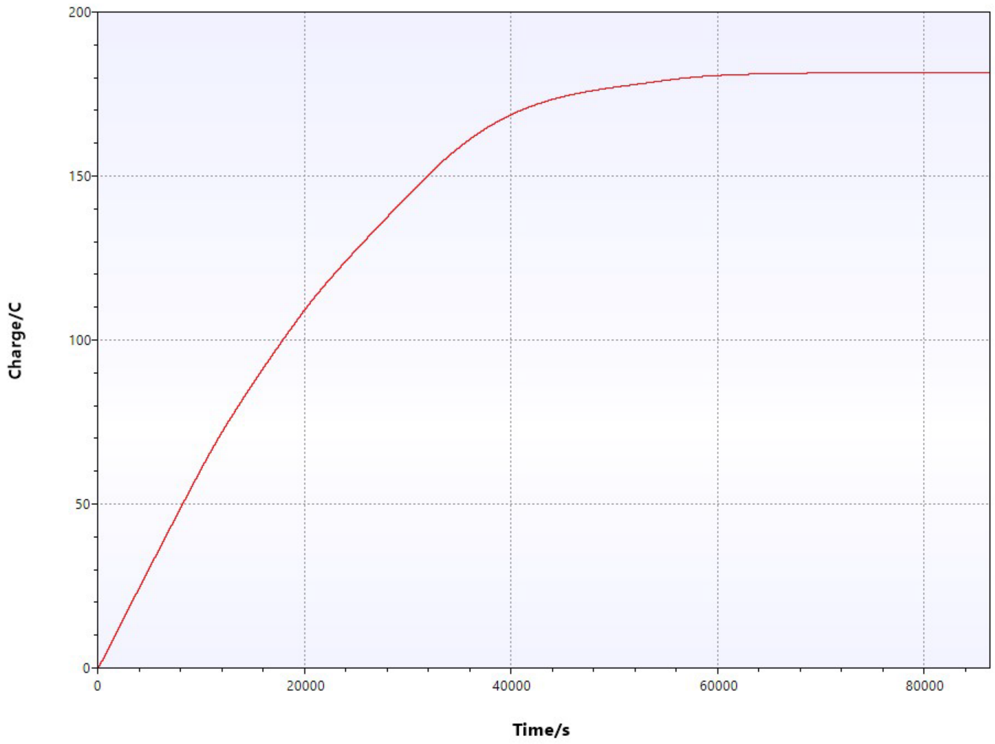

Figure S22. Current-time relationship for oxidation of (4-methoxyphenyl)methanol (**2a**).

S5. Fluorescence quenching study

The relative fluorescence intensities were measured on a Varian Eclipse spectrometer. Fluorescence quenching by substrate **2b** was measured in acetonitrile-water mixture (9:1) containing **1a** (5×10^{-3} mol/dm^3) at 25 °C. Stern-Volmer plot ($I_0/I = 1 + K_{SV}[Q]$) was constructed, and the constant K_S was evaluated as the slope of the dependence by using Origin 2018b 64-bit software.

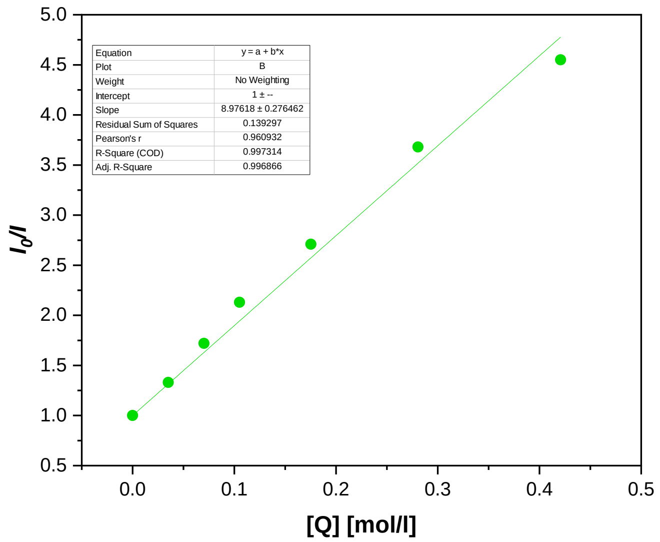

Figure S23. Stern-Volmer plot of fluorescence quenching of flavinium salt **1a** with (4-chlophenyl)methanol (**2b**) in mixture acetonitrile-water. K_S = 8.98 dm^3/mol.

S6. Theoretical calculations

Theoretical Calculations: Input structures were optimized[11] at B3LYP/6-311+G(d) level[12] either in vacuum, or in frame of the implicit solvation model (CPCM)[13-14]for water and acetonitrile. Identity of minima was verified by vibrational frequency calculations. The spin density localization was depicted using the MaSK program[15] at the isosurface level 0.025. Free energy based estimations of the dissociation equilibria in the solution (water, acetonitrile) state include the correction for the standard volume of 1 liter.[16]

Table S8. Hirshfeld[17] spin density at the *para*-located moiety in the cation radicals with respect to the CH_2OH group.

**Alcohol**	***para*-C atom**	**neighboring atom/group**
Benzyl alcohol$^{\bullet+}$ (**2i**$^{\bullet+}$)	0.293	0.014 (–H)
4-Chlorobenzyl alcohol$^{\bullet+}$ (**2b**$^{\bullet+}$)	0.223	0.186 (–Cl)
4-Methylbenzyl alcohol$^{\bullet+}$ (**2c**$^{\bullet+}$)	0.263	0.084 (–CH_3)
4-Methoxybenzyl alcohol$^{\bullet+}$ (**2a**$^{\bullet+}$)	0.166	0.191 (–O–)
4-Methylsulfanylbenzyl alcohol$^{\bullet+}$ (**2g**$^{\bullet+}$)	0.115	0.401 (–S–)

Table S9. Equilibrium constants for reactions of type at 298.15 K

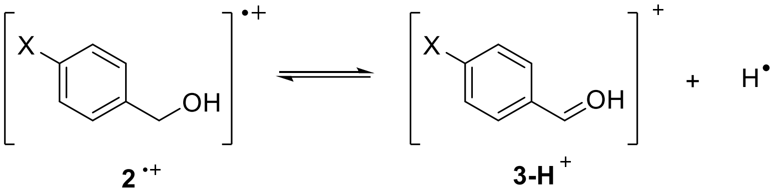

**Alcohol**	**log *K***		
	**gas phase**	**water**	**acetonitrile**
Benzyl alcohol (**2i**)	−17.0	−17.2	−17.3
4-Chlorobenzyl alcohol (**2b**)	−19.6	−18.8	−18.9
4-Methylbenzyl alcohol (**2c**)	−19.3	−19.6	−19.6
4-Methoxybenzyl alcohol (**2a**)	−22.3	−22.9	−22.9
4-Methylsulfanylbenzyl alcohol (**2g**)	−26.5	−27.3	−27.3

Table S10: Summary of thermochemistry details on the cation radicals (vacuum)

**Alcohol**	Number of imaginary frequencies	Total energy (HF) / hartree	Sum of electronic and thermal Free Energies / hartree
Benzyl alcohol$^{\bullet+}$ (**2i**$^{\bullet+}$)	0	-346.5420666	-346.443988
4-Chlorobenzyl alcohol$^{\bullet+}$ (**2b**$^{\bullet+}$)	0	-806.1713107	-806.083992
4-Methylbenzyl alcohol$^{\bullet+}$ (**2c**$^{\bullet+}$)	0	-385.8808754	-385.759124
4-Methoxybenzyl alcohol$^{\bullet+}$ (**2a**$^{\bullet+}$)	0	-461.1283334	-460.999797
4-Methylsulfanylbenzyl alcohol$^{\bullet+}$ (**2g**$^{\bullet+}$)	0	-784.1175035	-783.993744

Table S11: Summary of thermochemistry details on the cation radicals (water)

**Alcohol**	Number of imaginary frequencies	Total energy (HF) / hartree	Sum of electronic and thermal Free Energies / hartree
Benzyl alcohol$^{\bullet+}$ (**2i**$^{\bullet+}$)	0	-346.6208175	-346.522718
4-Chlorobenzyl alcohol$^{\bullet+}$ (**2b**$^{\bullet+}$)	0	-806.2481204	-806.160803
4-Methylbenzyl alcohol$^{\bullet+}$ (**2c**$^{\bullet+}$)	0	-385.9562551	-385.834007
4-Methoxybenzyl alcohol$^{\bullet+}$ (**2a**$^{\bullet+}$)	0	-461.2022457	-461.074094
4-Methylsulfanylbenzyl alcohol$^{\bullet+}$ (**2g**$^{\bullet+}$)	0	784.1907588	-784.067151

Table S12: Summary of thermochemistry details on the cation radicals (acetonitrile)

**Alcohol**	Number of imaginary frequencies	Total energy (HF) / hartree	Sum of electronic and thermal Free Energies / hartree
Benzyl alcohol$^{\bullet+}$ (**2i**$^{\bullet+}$)	0	-346.6195842	-346.521484
4-Chlorobenzyl alcohol$^{\bullet+}$ (**2b**$^{\bullet+}$)	0	-806.2469117	-806.159587
4-Methylbenzyl alcohol$^{\bullet+}$ (**2c**$^{\bullet+}$)	0	-385.9550727	-385.832827
4-Methoxybenzyl alcohol$^{\bullet+}$ (**2a**$^{\bullet+}$)	0	-461.2010859	-461.072922
4-Methylsulfanylbenzyl alcohol$^{\bullet+}$ (**2g**$^{\bullet+}$)	0	-784.1896039	-784.065986

Table S13: Summary of thermochemistry details on the cations (vacuum)

**Alcohol**	Number of imaginary frequencies	Total energy (HF) / hartree	Sum of electronic and thermal Free Energies / hartree
Benzyl alcohol^{+} (**2i**$^{+}$)	0	-345.9867993	-345.894322
4-Chlorobenzyl alcohol^{+} (**2b**$^{+}$)	0	-805.6092282	-805.528627
4-Methylbenzyl alcohol^{+} (**2c**$^{+}$)	0	-385.3205270	-385.204282
4-Methoxybenzyl alcohol^{+} (**2a**$^{+}$)	0	-460.5600205	-460.438419
4-Methylsulfanylbenzyl alcohol^{+} (**2g**$^{+}$)	0	-783.5397320	-783.423320

Table S14: Summary of thermochemistry details on the cations (water)

**Alcohol**	Number of imaginary frequencies	Total energy (HF) / hartree	Sum of electronic and thermal Free Energies / hartree
Benzyl alcohol^{+} (**2i**$^{+}$)	0	-346.0681908	-345.975417
4-Chlorobenzyl alcohol^{+} (**2b**$^{+}$)	0	-805.6909206	-805.610107
4-Methylbenzyl alcohol^{+} (**2c**$^{+}$)	0	-385.3982643	-385.281568
4-Methoxybenzyl alcohol^{+} (**2a**$^{+}$)	0	-460.6356465	-460.514465
4-Methylsulfanylbenzyl alcohol^{+} (**2g**$^{+}$)	0	-783.6142066	-783.497954

Table S15: Summary of thermochemistry details on the cations (acetonitrile)

**Alcohol**	Number of imaginary frequencies	Total energy (HF) / hartree	Sum of electronic and thermal Free Energies / hartree
Benzyl alcohol$^+$ (**2i**$^+$)	0	-346.0669009	-345.974132
4-Chlorobenzyl alcohol$^+$ (**2b**$^+$)	0	-805.6895957	-805.608785
4-Methylbenzyl alcohol$^+$ (**2c**$^+$)	0	-385.3970305	-385.280344
4-Methoxybenzyl alcohol$^+$ (**2a**$^+$)	0	-460.6344463	-460.513253
4-Methylsulfanylbenzyl alcohol$^+$ (**2g**$^+$)	0	-783.6130190	-783.496762

Table S16: Bond dissociation energies (BDE) of the benzylic C-H bond in cation radicals (HF based, in vacuum)

**Alcohol**	BDE / kJ.mol^{-1}
Benzyl alcohol (**2i**)	139,4
4-Chlorobenzyl alcohol (**2b**)	157,3
4-Methylbenzyl alcohol (**2c**)	152,8
4-Methoxybenzyl alcohol (**2a**)	173,7
4-Methylsulfanylbenzyl alcohol (**2g**)	198,5

S7. Cartesian coordinates of the optimized structures

A. Cation radicals

Benzyl alcohol (**2i**$^{\bullet+}$), vacuum

```
16

C  -0.437717 0.255245  -0.000001
C  0.532509  1.318060  -0.000002
C  1.872391  1.027513  0.000001
C  2.284838  -0.324454 0.000002
C  1.331178  -1.384645 0.000000
C  -0.011128 -1.101543 -0.000002
H  0.190429  2.348089  -0.000003
H  2.612473  1.818639  0.000002
H  3.343280  -0.562500 0.000003
H  1.678976  -2.411192 -0.000001
H  -0.758930 -1.884025 -0.000004
C  -1.871484 0.615820  0.000000
H  -2.054777 1.275418  -0.873795
O  -2.691757 -0.504448 0.000001
H  -2.054774 1.275418  0.873797
H  -3.626149 -0.260237 0.000004
```

Benzyl alcohol (**2i**$^{\bullet+}$), water (CPCM)

```
16

C  -0.437746 0.244993  0.000007
C  0.528072  1.313916  0.000001
C  1.869664  1.030897  -0.000007
C  2.288476  -0.314860 -0.000005
C  1.339707  -1.380839 0.000002
C  -0.003303 -1.106381 0.000009
H  0.177487  2.339768  0.000003
H  2.604139  1.826038  -0.000014
H  3.346489  -0.549440 -0.000005
H  1.694875  -2.403875 0.000002
H  -0.741455 -1.896648 0.000011
C  -1.865680 0.609800  0.000009
H  -2.048638 1.268425  -0.871354
O  -2.708661 -0.502974 -0.000017
H  -2.048635 1.268378  0.871411
H  -3.630107 -0.214019 -0.000011
```

Benzyl alcohol (**2i**$^{\bullet+}$), acetonitrile (CPCM)

```
16

C  -0.437744 0.245193  0.000006
C  0.528149  1.314000  0.000001
C  1.869700  1.030837  -0.000007
C  2.288394  -0.315046 -0.000004
C  1.339543  -1.380910 0.000002
C  -0.003450 -1.106285 0.000008
H  0.177714  2.339926  0.000003
H  2.604291  1.825890  -0.000014
H  3.346414  -0.549683 -0.000005
```

```
H  1.694565  -2.404011 0.000002
H  -0.741785 -1.896399 0.000011
C  -1.865782 0.609900  0.000009
H  -2.048746 1.268545  -0.871398
O  -2.708319 -0.503005 -0.000017
H  -2.048742 1.268500  0.871454
H  -3.630020 -0.214868 -0.000011
```

4-Chlorobenzyl alcohol (**2b$^{•+}$**), vacuum

```
16

C  -0.762532 -1.022779 -0.000008
C  -1.332792 0.278960  -0.000004
C  -0.484812 1.428680  -0.000007
C  0.876439  1.289913  -0.000005
C  1.430941  -0.019668 -0.000008
C  0.599778  -1.176898 -0.000006
C  -2.805341 0.472351  0.000011
O  -3.472190 -0.752011 -0.000003
H  -0.926889 2.419736  -0.000011
H  1.534169  2.150415  -0.000005
Cl 3.115927  -0.210314 0.000008
H  1.056327  -2.159435 -0.000004
H  -1.422476 -1.880743 -0.000009
H  -3.067378 1.094331  -0.877419
H  -3.067368 1.094306  0.877458
H  -4.429713 -0.630539 0.000041
```

4-Chlorobenzyl alcohol (**2b$^{•+}$**), water (CPCM)

```
16

C  -0.756230 -1.030939 -0.000009
C  -1.330896 0.267416  0.000000
C  -0.486367 1.421430  -0.000005
C  0.874425  1.288223  -0.000009
C  1.428855  -0.017070 -0.000008
C  0.605380  -1.179283 -0.000011
C  -2.796776 0.470992  0.000010
O  -3.494113 -0.744367 0.000001
H  -0.935467 2.407760  -0.000008
H  1.527512  2.150825  -0.000014
Cl 3.119108  -0.205313 0.000010
H  1.066028  -2.158585 -0.000013
H  -1.406569 -1.894813 -0.000010
H  -3.054796 1.094760  -0.874708
H  -3.054802 1.094746  0.874730
H  -4.444188 -0.574049 0.000039
```

4-Chlorobenzyl alcohol (**2b$^{•+}$**), acetonitrile (CPCM)

```
16

C  -0.756345 -1.030775 -0.000001
C  -1.330920 0.267646  0.000000
C  -0.486331 1.421571  -0.000001
C  0.874461  1.288255  -0.000001
C  1.428904  -0.017133 0.000000
```

```
C  0.605276  -1.179240 -0.000001
C  -2.796933 0.471019  0.000000
O  -3.493672 -0.744519 0.000001
H  -0.935299 2.407990  0.000000
H  1.527651  2.150803  -0.000001
Cl 3.119020  -0.205407 0.000001
H  1.065842  -2.158603 -0.000002
H  -1.406867 -1.894531 -0.000001
H  -3.055029 1.094732  -0.874773
H  -3.055030 1.094733  0.874773
H  -4.443904 -0.575105 0.000002
```

4-Methylbenzyl alcohol (**2c**$^{•+}$), vacuum

```
19

C  0.403714  -1.039786 -0.002850
C  0.946287  0.273669  -0.000455
C  0.066708  1.404513  -0.001092
C  -1.289685 1.226672  -0.004314
C  -1.845830 -0.086168 -0.007001
C  -0.954480 -1.212175 -0.006187
C  2.411307  0.506763  0.002592
O  3.115406  -0.696505 0.002898
H  0.487631  2.404992  -0.000258
H  -1.954973 2.082497  -0.005991
C  -3.311433 -0.303853 0.003540
H  -1.377327 -2.211276 -0.008478
H  1.082063  -1.883180 -0.003012
H  2.653651  1.137047  0.880227
H  2.656510  1.138050  -0.873619
H  4.068499  -0.544601 0.006841
H  -3.605519 -1.051125 -0.742217
H  -3.613841 -0.729768 0.972605
H  -3.879467 0.611591  -0.154674
```

4-Methylbenzyl alcohol (**2c**$^{•+}$), water (CPCM)

```
19

C  0.396472  -1.045388 0.000003
C  0.943942  0.263019  0.000002
C  0.068206  1.398468  0.000002
C  -1.288726 1.228328  0.000002
C  -1.847660 -0.079294 -0.000001
C  -0.961687 -1.210990 0.000002
C  2.404050  0.504963  -0.000002
O  3.134371  -0.692740 -0.000002
H  0.497395  2.394026  0.000000
H  -1.948661 2.087270  0.000002
C  -3.312027 -0.299165 -0.000005
H  -1.391927 -2.205827 0.000004
H  1.064551  -1.895729 0.000004
H  2.646156  1.134234  0.875050
H  2.646150  1.134230  -0.875059
H  4.079207  -0.495555 -0.000006
H  -3.601886 -0.899851 -0.871785
H  -3.601897 -0.899773 0.871827
```

```
H  -3.879481 0.629252  -0.000045
```

4-Methylbenzyl alcohol (**2c**$^{•+}$), acetonitrile (CPCM)

```
19

C  0.396605  -1.045266 0.000002
C  0.943988  0.263232  0.000002
C  0.068187  1.398599  0.000002
C  -1.288737 1.228325  -0.000001
C  -1.847616 -0.079390 -0.000001
C  -0.961549 -1.210985 0.000000
C  2.404185  0.504980  -0.000001
O  3.133965  -0.692840 0.000000
H  0.497237  2.394241  0.000004
H  -1.948775 2.087205  -0.000003
C  -3.311995 -0.299271 0.000000
H  -1.391661 -2.205896 -0.000001
H  1.064873  -1.895475 0.000003
H  2.646328  1.134279  0.875086
H  2.646324  1.134276  -0.875090
H  4.078978  -0.496526 -0.000007
H  -3.601951 -0.899913 -0.871796
H  -3.601951 -0.899915 0.871795
H  -3.879526 0.629105  0.000001
```

4-Methoxybenzyl alcohol (**2c**$^{•+}$), vacuum

```
20

C  -1.386674 0.275299  0.000003
C  -0.665083 1.500978  0.000002
C  0.699991  1.499935  -0.000005
C  1.408181  0.252069  -0.000017
C  0.692590  -0.982404 -0.000013
C  -0.678106 -0.958472 -0.000006
H  -1.205061 2.442126  0.000003
H  1.280053  2.415105  -0.000005
O  2.712343  0.357127  -0.000015
H  1.221584  -1.926638 -0.000023
H  -1.248836 -1.878170 -0.000011
C  -2.878002 0.301800  0.000017
H  -3.211617 0.878533  -0.880368
O  -3.387568 -1.003654 -0.000013
H  -3.211594 0.878480  0.880446
H  -4.352090 -0.997874 0.000036
C  3.589633  -0.800919 0.000026
H  4.592627  -0.387133 0.000123
H  3.420702  -1.390995 0.900664
H  3.420848  -1.390927 -0.900686
```

4-Methoxybenzyl alcohol (**2a**$^{•+}$), water (CPCM)

```
20

C  -1.381631 0.266228  -0.000007
C  -0.664371 1.495480  -0.000006
C  0.699361  1.499696  -0.000001
C  1.411726  0.256066  0.000002
```

```
C  0.701279  -0.981946 -0.000003
C  -0.668881 -0.961859 -0.000007
H  -1.208725 2.433025  -0.000009
H  1.272597  2.418255  0.000001
O  2.715553  0.362044  0.000011
H  1.232254  -1.924033 0.000001
H  -1.227297 -1.887950 -0.000010
C  -2.870450 0.302397  0.000003
H  -3.202355 0.877654  -0.878829
O  -3.409861 -1.000355 0.000007
H  -3.202341 0.877653  0.878841
H  -4.372959 -0.946321 -0.000001
C  3.583140  -0.803183 -0.000003
H  4.588983  -0.397763 -0.000047
H  3.406671  -1.390635 0.899494
H  3.406605  -1.390668 -0.899464
```

4-Methoxybenzyl alcohol (**2a**$^{•+}$), acetonitrile (CPCM)

```
20

C  -1.381725 0.266417  -0.000004
C  -0.664384 1.495600  -0.000005
C  0.699372  1.499712  -0.000001
C  1.411654  0.256001  0.000001
C  0.701113  -0.981941 -0.000002
C  -0.669052 -0.961778 -0.000004
H  -1.208655 2.433211  -0.000008
H  1.272738  2.418205  -0.000002
O  2.715489  0.361952  0.000009
H  1.232045  -1.924069 0.000000
H  -1.227703 -1.887743 -0.000006
C  -2.870585 0.302376  0.000004
H  -3.202530 0.877655  -0.878856
O  -3.409386 -1.000441 0.000003
H  -3.202519 0.877649  0.878872
H  -4.372531 -0.947327 -0.000003
C  3.583215  -0.803167 -0.000003
H  4.589018  -0.397630 -0.000042
H  3.406861  -1.390662 0.899512
H  3.406802  -1.390693 -0.899485
```

4-Methylsulfanylbenzyl alcohol (**2g**$^{•+}$), vacuum

```
20

C  -1.756310 0.261521  -0.000001
C  -1.057908 1.491226  -0.000035
C  0.313746  1.515096  -0.000023
C  1.045905  0.288340  0.000016
C  0.345909  -0.946403 0.000057
C  -1.030157 -0.951625 0.000048
H  -1.609766 2.425584  -0.000073
H  0.846063  2.460183  -0.000055
S  2.757193  0.454387  0.000051
H  0.882946  -1.886096 0.000101
H  -1.579284 -1.884248 0.000083
C  -3.252900 0.262080  -0.000009
```

```
H  -3.599307 0.825541  -0.881955
O  -3.732016 -1.058582 -0.000027
H  -3.599303 0.825513  0.881960
H  -4.696221 -1.072430 0.000000
C  3.440169  -1.230422 -0.000098
H  4.521434  -1.099109 -0.000293
H  3.142046  -1.767688 0.899647
H  3.141714  -1.767660 -0.899747
```

4-Methylsulfanylbenzyl alcohol (**2g**$^{\bullet+}$), water (CPCM)

```
20

C  -1.751219 0.253608  0.000000
C  -1.056330 1.484373  0.000004
C  0.315402  1.512179  0.000001
C  1.049618  0.288769  -0.000004
C  0.354294  -0.947373 -0.000009
C  -1.022680 -0.954295 -0.000007
H  -1.610793 2.416354  0.000009
H  0.844780  2.457866  0.000005
S  2.758109  0.453994  -0.000006
H  0.893271  -1.884826 -0.000015
H  -1.559543 -1.893181 -0.000011
C  -3.247478 0.265849  0.000005
H  -3.593223 0.826258  -0.880715
O  -3.757605 -1.053836 0.000001
H  -3.593215 0.826250  0.880734
H  -4.721510 -1.019649 0.000000
C  3.438752  -1.227401 0.000015
H  4.518850  -1.094558 0.000016
H  3.135155  -1.760987 0.899072
H  3.135162  -1.761010 -0.899031
```

4-Methylsulfanylbenzyl alcohol (**2g**$^{\bullet+}$), acetonitrile (CPCM)

```
20

C  -1.751306 0.253773  -0.000008
C  -1.056354 1.484530  0.000009
C  0.315367  1.512266  0.000010
C  1.049546  0.288787  -0.000004
C  0.354138  -0.947337 -0.000017
C  -1.022806 -0.954230 -0.000019
H  -1.610781 2.416545  0.000022
H  0.844803  2.457939  0.000022
S  2.758084  0.453994  -0.000009
H  0.893076  -1.884831 -0.000027
H  -1.559902 -1.892991 -0.000031
C  -3.247554 0.265755  -0.000010
H  -3.593308 0.826195  -0.880770
O  -3.757055 -1.053949 0.000030
H  -3.593308 0.826247  0.880719
H  -4.720986 -1.020669 -0.000013
C  3.438695  -1.227498 0.000022
H  4.518818  -1.094738 0.000010
H  3.135167  -1.761122 0.899095
H  3.135157  -1.761166 -0.899022
```

B. Cations

Benzyl alcohol (**2i**$^+$), vacuum

```
15

C   -0.009950 1.117412  0.000015
C   -0.482353 -0.225909 -0.000001
C   0.438779  -1.310633 0.000006
C   1.795603  -1.054671 -0.000003
C   2.245633  0.271565  -0.000018
C   1.347303  1.352846  0.000005
C   -1.846252 -0.529725 0.000006
O   -2.748544 0.399822  -0.000013
H   0.073379  -2.332445 0.000015
H   2.507414  -1.871233 -0.000004
H   3.312304  0.469875  -0.000005
H   1.725123  2.368280  0.000010
H   -0.717266 1.938101  0.000010
H   -2.183772 -1.565762 0.000027
H   -3.661413 0.069305  -0.000017
```

Benzyl alcohol (**2i**$^+$), water (CPCM)

```
15

C   -0.010367 1.111605  0.000013
C   -0.480507 -0.227190 -0.000012
C   0.435242  -1.308771 0.000001
C   1.795049  -1.052231 -0.000010
C   2.246890  0.270850  -0.000017
C   1.348228  1.349437  0.000012
C   -1.853418 -0.529174 0.000023
O   -2.740464 0.404027  -0.000031
H   0.067354  -2.328482 0.000019
H   2.504928  -1.870083 -0.000011
H   3.312943  0.469359  0.000012
H   1.723617  2.365429  0.000036
H   -0.717637 1.931545  0.000015
H   -2.200068 -1.560118 0.000098
H   -3.654133 0.072977  0.000017
```

Benzyl alcohol (**2i**$^+$), acetonitrile (CPCM)

```
15

C   -0.010346 1.111736  0.000013
C   -0.480544 -0.227147 -0.000011
C   0.435303  -1.308803 0.000001
C   1.795044  -1.052288 -0.000010
C   2.246851  0.270849  -0.000017
C   1.348215  1.349498  0.000012
C   -1.853238 -0.529168 0.000023
O   -2.740644 0.403937  -0.000031
H   0.067450  -2.328548 0.000019
H   2.504961  -1.870106 -0.000011
H   3.312910  0.469345  0.000011
H   1.723674  2.365463  0.000035
```

```
H  -0.717596 1.931703  0.000015
H  -2.199700 -1.560211 0.000097
H  -3.654254 0.072800  0.000017
```

4-Chlorobenzyl alcohol (**2b**$^{+}$), vacuum

```
15

C  0.584086  -1.178619 0.000005
C  -0.783312 -1.058859 0.000001
C  -1.382657 0.234491  0.000000
C  -0.557121 1.394780  0.000004
C  0.812891  1.271522  0.000003
C  1.381897  -0.014469 0.000010
C  -2.765474 0.408601  0.000000
O  -3.576524 -0.605803 -0.000003
Cl 3.087774  -0.174941 -0.000005
H  -1.007863 2.381829  -0.000015
H  1.452743  2.144886  0.000004
H  1.058137  -2.152208 -0.000001
H  -1.408772 -1.943556 0.000009
H  -3.200162 1.407314  -0.000005
H  -4.515918 -0.362507 -0.000016
```

4-Chlorobenzyl alcohol (**2b**$^{+}$), water (CPCM)

```
15

C  0.586055  -1.176618 0.000000
C  -0.785170 -1.057246 -0.000003
C  -1.381165 0.230246  -0.000007
C  -0.565388 1.388636  0.000002
C  0.809790  1.268195  0.000001
C  1.372637  -0.012515 0.000006
C  -2.774722 0.405201  -0.000002
O  -3.574840 -0.603904 0.000006
Cl 3.098562  -0.171586 0.000000
H  -1.019166 2.372843  -0.000011
H  1.444250  2.144544  0.000005
H  1.058317  -2.150209 -0.000008
H  -1.407789 -1.943019 -0.000001
H  -3.211176 1.400951  -0.000006
H  -4.513489 -0.352304 0.000003
```

4-Chlorobenzyl alcohol (**2b**$^{+}$), acetonitrile (CPCM)

```
15

C  0.586043  -1.176670 0.000000
C  -0.785094 -1.057326 -0.000003
C  -1.381183 0.230282  -0.000007
C  -0.565214 1.388731  0.000002
C  0.809846  1.268248  0.000001
C  1.372844  -0.012556 0.000006
C  -2.774460 0.405256  -0.000002
O  -3.574930 -0.603892 0.000006
Cl 3.098295  -0.171625 0.000000
H  -1.018952 2.372984  -0.000011
H  1.444420  2.144526  0.000005
```

```
H  1.058372  -2.150239 -0.000008
H  -1.407738 -1.943096 -0.000001
H  -3.210824 1.401082  -0.000005
H  -4.513539 -0.352276 0.000003
```

4-Methylbenzyl alcohol (**2c**$^+$), vacuum

```
18

C  1.223614  1.215169  -0.006306
C  1.797248  -0.072713 -0.007858
C  0.947866  -1.207348 -0.008077
C  -0.417376 -1.071544 -0.003096
C  -0.993665 0.232763  -0.000222
C  -0.143620 1.374789  -0.001510
C  3.280638  -0.252095 0.006987
C  -2.370390 0.436970  0.003174
O  -3.207152 -0.558173 0.004155
H  -0.575540 2.370483  -0.000879
H  1.868570  2.086344  -0.009912
H  1.390045  -2.197648 -0.012706
H  -1.060288 -1.943735 -0.003593
H  -2.782480 1.445279  0.005494
H  -4.139278 -0.289645 0.007146
H  3.593285  -1.034974 -0.688668
H  3.812245  0.668349  -0.232870
H  3.604760  -0.575018 1.004195
```

4-Methylbenzyl alcohol (**2c**$^+$), water (CPCM)

```
18

C  1.796682  -0.069393 -0.005325
C  0.952212  -1.204144 -0.005028
C  -0.415312 -1.069821 -0.001706
C  -0.991503 0.229342  0.000080
C  -0.149241 1.369978  -0.000730
C  1.222100  1.213397  -0.004080
C  -2.376283 0.433629  0.001953
O  -3.204263 -0.558137 0.002477
C  3.283302  -0.248570 0.004497
H  -0.585156 2.362736  -0.000203
H  1.862783  2.087373  -0.006259
H  1.395939  -2.193440 -0.008079
H  -1.055525 -1.943366 -0.002009
H  -2.791310 1.438415  0.003213
H  -4.134780 -0.280221 0.004035
H  3.604631  -0.659169 0.968089
H  3.595124  -0.965119 -0.760060
H  3.810660  0.691381  -0.156505
```

4-Methylbenzyl alcohol (**2c**$^+$), acetonitrile (CPCM)

```
18

C  1.796689  -0.069422 -0.005345
C  0.952153  -1.204198 -0.005050
C  -0.415316 -1.069886 -0.001714
C  -0.991541 0.229372  0.000078
```

```
C  -0.149146 1.370060  -0.000735
C  1.222110  1.213455  -0.004095
C  -2.376122 0.433669  0.001961
O  -3.204359 -0.558123 0.002487
C  3.283230  -0.248617 0.004509
H  -0.585011 2.362861  -0.000207
H  1.862862  2.087379  -0.006285
H  1.395890  -2.193490 -0.008109
H  -1.055549 -1.943425 -0.002017
H  -2.791062 1.438530  0.003230
H  -4.134863 -0.280264 0.004057
H  3.604524  -0.658709 0.968347
H  3.595039  -0.965660 -0.759590
H  3.810700  0.691175  -0.156977
```

4-Methoxybenzyl alcohol (**2a⁺**), vacuum

```
19

C  -0.690957 -0.999854 0.000001
C  -1.433267 0.220636  -0.000003
C  -0.724547 1.464125  -0.000006
C  0.639766  1.484454  0.000000
C  1.365114  0.262523  0.000005
C  0.677216  -0.983264 0.000002
C  -2.817215 0.239751  -0.000003
O  -3.519902 -0.863181 0.000003
O  2.673983  0.384089  0.000016
C  3.544288  -0.770729 -0.000011
H  -1.277485 2.397787  -0.000013
H  1.198565  2.412248  0.000000
H  1.227121  -1.914811 0.000000
H  -1.221069 -1.944959 0.000004
H  -3.360223 1.183333  -0.000004
H  -4.477079 -0.713293 0.000002
H  4.549985  -0.362269 -0.000018
H  3.382583  -1.365591 0.899683
H  3.382559  -1.365565 -0.899719
```

4-Methoxybenzyl alcohol (**2a⁺**), water (CPCM)

```
19

C  -0.685654 -0.998431 0.000000
C  -1.429386 0.215070  -0.000008
C  -0.730435 1.458108  -0.000007
C  0.637308  1.483281  0.000000
C  1.364489  0.268086  0.000009
C  0.685266  -0.977259 0.000010
C  -2.819916 0.235162  -0.000006
O  -3.518454 -0.861434 0.000005
O  2.683154  0.392085  0.000027
C  3.532725  -0.774828 -0.000022
H  -1.288758 2.387294  -0.000012
H  1.186804  2.416373  -0.000005
H  1.235709  -1.907789 0.000023
H  -1.208984 -1.946680 -0.000006
H  -3.360655 1.178038  -0.000012
```

```
H  -4.475865 -0.703152 0.000006
H  4.546364  -0.385888 -0.000052
H  3.360734  -1.369278 0.897450
H  3.360668  -1.369249 -0.897501
```

4-Methoxybenzyl alcohol (**2a**$^{+}$), acetonitrile (CPCM)

```
19

C  -0.685725 -0.998488 0.000002
C  -1.429455 0.215152  -0.000008
C  -0.730303 1.458216  -0.000008
C  0.637352  1.483307  0.000001
C  1.364515  0.267977  0.000010
C  0.685123  -0.977396 0.000012
C  -2.819810 0.235252  -0.000007
O  -3.518534 -0.861447 0.000004
O  2.682935  0.391935  0.000028
C  3.532929  -0.774736 -0.000025
H  -1.288527 2.387485  -0.000014
H  1.187032  2.416288  -0.000004
H  1.235571  -1.907933 0.000026
H  -1.209166 -1.946685 -0.000002
H  -3.360567 1.178151  -0.000014
H  -4.475911 -0.703187 0.000004
H  4.546401  -0.385393 -0.000062
H  3.361136  -1.369179 0.897495
H  3.361058  -1.369154 -0.897546
```

4-Methylsulfanylbenzyl alcohol (**2g**$^{+}$), vacuum

```
19

C  -1.041201 -1.001007 0.000007
C  -1.806094 0.205334  0.000003
C  -1.117042 1.457903  0.000003
C  0.248709  1.500169  0.000004
C  1.005573  0.294356  0.000001
C  0.326319  -0.955883 0.000004
C  -3.190787 0.201205  -0.000004
O  -3.873479 -0.916253 -0.000005
S  2.721026  0.478076  -0.000004
C  3.395892  -1.214760 -0.000001
H  -1.683288 2.383618  0.000001
H  0.763685  2.454307  0.000007
H  0.886138  -1.881436 0.000005
H  -1.552594 -1.956403 0.000013
H  -3.751072 1.134352  -0.000008
H  -4.832823 -0.782599 -0.000009
H  4.477307  -1.082336 -0.000006
H  3.107929  -1.756300 0.900027
H  3.107922  -1.756305 -0.900024
```

4-Methylsulfanylbenzyl alcohol (**2g**$^{+}$), water (CPCM)

```
19

C  -1.039577 -0.998046 -0.000005
C  -1.803396 0.202787  -0.000002
```

```
C  -1.123735 1.454454  0.000002
C  0.245907  1.500341  0.000002
C  1.001187  0.299685  -0.000006
C  0.330828  -0.950221 -0.000009
C  -3.195456 0.197382  0.000001
O  -3.868188 -0.915954 0.000005
S  2.728434  0.479197  -0.000002
C  3.387518  -1.219169 0.000010
H  -1.694234 2.376344  0.000001
H  0.756078  2.456544  0.000010
H  0.891199  -1.874650 -0.000014
H  -1.545785 -1.955590 -0.000005
H  -3.757288 1.127875  0.000002
H  -4.828689 -0.780672 0.000005
H  4.469831  -1.097816 0.000019
H  3.089887  -1.757409 0.898144
H  3.089904  -1.757417 -0.898126
```

4-Methylsulfanylbenzyl alcohol (**2g**$^{+}$), acetonitrile (CPCM)

```
19

C  -1.039557 -0.998159 -0.000005
C  -1.803434 0.202788  -0.000002
C  -1.123563 1.454495  0.000002
C  0.245974  1.500325  0.000002
C  1.001318  0.299560  -0.000006
C  0.330767  -0.950375 -0.000008
C  -3.195285 0.197472  0.000001
O  -3.868396 -0.915904 0.000004
S  2.728233  0.479180  -0.000002
C  3.387665  -1.219043 0.000009
H  -1.693989 2.376454  0.000001
H  0.756250  2.456476  0.000010
H  0.891150  -1.874807 -0.000014
H  -1.545843 -1.955674 -0.000004
H  -3.757014 1.128053  0.000002
H  -4.828838 -0.780436 0.000004
H  4.469948  -1.097417 0.000019
H  3.090226  -1.757337 0.898184
H  3.090244  -1.757345 -0.898167
```

S8. References

1. Neveselý, T.; Svobodová, E.; Chudoba, J.; Sikorski, M.; Cibulka, R., Efficient Metal-Free Aerobic Photooxidation of Sulfides to Sulfoxides Mediated by a Vitamin B2 Derivative and Visible Light. *Adv. Synth. Catal.* **2016,** *358* (10), 1654-1663.

2. Pokluda, A.; Anwar, Z.; Boguschová, V.; Anusiewicz, I.; Skurski, P.; Sikorski, M.; Cibulka, R., Robust Photocatalytic Method Using Ethylene-Bridged Flavinium Salts for the Aerobic Oxidation of Unactivated Benzylic Substrates. *Adv. Synth. Catal.* **2021,** *363* (18), 4371-4379.

3. Tolba, A. H.; Vávra, F.; Chudoba, J.; Cibulka, R., Tuning Flavin-Based Photocatalytic Systems for Application in the Mild Chemoselective Aerobic Oxidation of Benzylic Substrates. *Eur. J. Org. Chem.* **2020,** *2020* (10), 1579-1585.

4. Pavlovska, T.; Weisheitelová, I.; Pramthaisong, C.; Sikorski, M.; Jahn, U.; Cibulka, R., Primary and Secondary Amines by Flavin-Photocatalyzed Consecutive Desulfonylation and Dealkylation of Sulfonamides. *Adv. Synth. Catal.* **2023,** *365* (24), 4662-4671.

5. Mojr, V.; Svobodová, E.; Straková, K.; Neveselý, T.; Chudoba, J.; Dvořáková, H.; Cibulka, R., Tailoring flavins for visible light photocatalysis: organocatalytic [2+2] cycloadditions mediated by a flavin derivative and visible light. *Chem. Commun.* **2015,** *51* (60), 12036-12039.

6. Hartman, T.; Reisnerová, M.; Chudoba, J.; Svobodová, E.; Archipowa, N.; Kutta, R. J.; Cibulka, R., Photocatalytic Oxidative [2+2] Cycloelimination Reactions with Flavinium Salts: Mechanistic Study and Influence of the Catalyst Structure. *ChemPlusChem* **2021,** *86* (3), 373-386.

7. Krejcik, M.; Daněk, M.; Hartl, F., Simple construction of an infrared optically transparent thin-layer electrochemical cell: Applications to the redox reactions of ferrocene, Mn2(CO)10 and Mn(CO)3(3,5-di-t-butyl-catecholate)−. *J. Electroanal. Chem.* **1991,** *317*, 179-187.

8. Bai, Y.; Cao, L.; Li, S.; Zhang, G. a.; Liu, Y.; Zhao, F.; Wu, J., Dual Photoredox/Nickel-Catalyzed Dehydrative Difluoroalkylation of Benzyl Alcohols for the Synthesis of Allylic gem-Difluorides. *Org. Lett.* **2023,** *25* (35), 6511-6516.

9. Hruzd, M.; Kleynemeyer, S. L.; Michon, C.; Bastin, S.; Pollet, E.; Ritleng, V.; Sortais, J.-B., Thioether–NHC bidentate manganese complexes as efficient phosphine-free catalysts for hydrogenation at room temperature. *Chem. Commun.* **2025,** *61* (14), 2969-2972.

10. Šimková, L.; Svobodová, E.; Liška, A.; Lušpai, K.; Cibulka, R.; Ludvík, J., Redox properties of flavin derivatives: A comparative study. *Electrochim. Acta* **2025,** *543*, 147520.

11. Frisch, M. J.; Trucks, G. W.; Schlegel, H. B.; Scuseria, G. E.; Robb, M. A.; Cheeseman, J. R.; Scalmani, G.; Barone, V.; Petersson, G. A.; Nakatsuji, H.; Li, X.; Caricato, M.; Marenich, A. V.; Bloino, J.; Janesko, B. G.; Gomperts, R.; Mennucci, B.; Hratchian, H. P.; Ortiz, J. V.; Izmaylov, A. F.; Sonnenberg, J. L.; Williams; Ding, F.; Lipparini, F.; Egidi, F.; Goings, J.; Peng, B.; Petrone, A.; Henderson, T.; Ranasinghe, D.; Zakrzewski, V. G.; Gao, J.; Rega, N.; Zheng, G.; Liang, W.; Hada, M.; Ehara, M.; Toyota, K.; Fukuda, R.; Hasegawa, J.; Ishida, M.; Nakajima, T.; Honda, Y.; Kitao, O.; Nakai, H.; Vreven, T.; Throssell, K.; Montgomery Jr., J. A.; Peralta, J. E.; Ogliaro, F.; Bearpark, M. J.; Heyd, J. J.; Brothers, E. N.; Kudin, K. N.; Staroverov, V. N.; Keith, T. A.; Kobayashi, R.; Normand, J.; Raghavachari, K.; Rendell, A. P.; Burant, J. C.; Iyengar, S. S.; Tomasi, J.; Cossi, M.; Millam, J. M.; Klene, M.; Adamo, C.; Cammi, R.; Ochterski, J. W.; Martin, R. L.; Morokuma, K.; Farkas, O.; Foresman, J. B.; Fox, D. J. *Gaussian 16 Rev. C.01*, Wallingford, CT, 2016.

12. Becke, A. D., Density-functional thermochemistry. III. The role of exact exchange. *The Journal of Chemical Physics* **1993,** *98* (7), 5648-5652.

13. Klamt, A.; Schüürmann, G., COSMO: a new approach to dielectric screening in solvents with explicit expressions for the screening energy and its gradient. *Journal of the Chemical Society, Perkin Transactions 2* **1993,** (5), 799-805.

14. Amovilli, C.; Barone, V.; Cammi, R.; Cancès, E.; Cossi, M.; Mennucci, B.; Pomelli, C. S.; Tomasi, J., Recent Advances in the Description of Solvent Effects with the Polarizable Continuum Model. In *Adv. Quantum Chem.*, Löwdin, P.-O., Ed. Academic Press: 1998; Vol. 32, pp 227-261.

15. Podolyan, Y.; Leszczynski, J., MaSK: A Visualization Tool for Teaching and Research in Computational Chemistry. *Int. J. Quantum Chem* **2009,** *109*, 8-16.

16. Jensen, J. H., Predicting accurate absolute binding energies in aqueous solution: thermodynamic considerations for electronic structure methods. *Physical Chemistry Chemical Physics* **2015,** *17* (19), 12441-12451.
17. Hirshfeld, F. L., Bonded-atom fragments for describing molecular charge densities. *Theoretica chimica acta* **1977,** *44* (2), 129-138.
